# Dual PARP and RAD51 Inhibitory Drug Conjugates Show Synergistic and Selective Effects on Breast Cancer Cells

**DOI:** 10.3390/biom11070981

**Published:** 2021-07-03

**Authors:** Matthews M. Malka, Julia Eberle, Kathrin Niedermayer, Darius P. Zlotos, Lisa Wiesmüller

**Affiliations:** 1 Department of Pharmaceutical Chemistry, The German University in Cairo, New Cairo City, Main Entrance of Al Tagamoa Al Khames, Cairo 11835, Egypt; Matthews.magdy@hotmail.com; 2 Department of Obstetrics and Gynecology, Ulm University, Prittwitzstrasse 43, 89075 Ulm, Germany; julia.eberle@uniklinik-ulm.de (J.E.); kathrin.niedermayer@uniklinik-ulm.de (K.N.)

**Keywords:** PARP inhibitor, Olaparib, RAD51 inhibitor, drug conjugates, anticancer drug hybrids, triple-negative breast cancer

## Abstract

The genetic principle of synthetic lethality has most successfully been exploited in therapies engaging Poly-ADP-ribose-polymerase (PARP) inhibitors to treat patients with homologous recombination (HR)-defective tumors. In this work, we went a step further following the idea of a local molecular cooperation and designed hybrid compounds **M1–M3**. The drug conjugates **M1–M3** combine **Olaparib**, the first PARP inhibitor approved for clinical use, with **Cpd 1**, an inhibitor of RAD51 that blocks its HR functions and yet permits RAD51 nucleoprotein filament formation on single-stranded DNA. While in **M2** and **M3**, the parental drugs are linked by -CO-(CH_2_)_n_-CO-spacers (n = 2 and 4, respectively), they are directly merged omitting the piperazine ring of **Olaparib** in **M1**. Monitoring anti-survival effects of **M1**–**M3** in six breast cancer cell lines of different molecular subtypes showed that in each cell line, at least one of the drug conjugates decreased viability by one to two orders of magnitude compared with parental drugs. While triple-negative breast cancer (TNBC) cells with frequent BRCA1 pathway dysfunction were sensitive to spacer-linked hybrid compounds **M1** and **M2** regardless of their HR capacities, non-TNBC cells were responsive to the merged drug conjugate **M1** only, suggesting different spatial requirements for dual inhibition in these two groups of cell lines. These results demonstrate that, depending on chemical linkage, dual PARP1-RAD51 inhibitory drugs can either sensitize non-TNBC and re-sensitize TNBC cells, or discriminate between these groups of cells.

## 1. Introduction

Application of drug conjugates (hybrid drugs) is an emerging approach to overcome drawbacks of current anticancer treatment, such as insufficient potency and efficacy, high toxicity, and development of resistance [1]. In anticancer drug conjugates, two drugs (pharmacophores) are combined in one molecule displaying simultaneous action at two different targets. In comparison to a combination of two single-target drugs, a hybrid drug may offer an advantage of pharmacokinetic simplicity and fewer drug–drug interactions [2]. A multitude of dual-acting hybrids with pharmacophores belonging to various pharmacological classes, such as DNA-alkylating agents, organoplatinum complexes, histone deacetylase inhibitors, topoisomerase inhibitors, have been reported as potential anticancer agents [1,2,3,4]. 

Poly-ADP-ribose-polymerase (PARP) inhibitors encompass an established class of anticancer agents used for the treatment of BRCA-mutated advanced tumors [5]. PARP inhibitor treatment of such homologous recombination (HR) deficient tumor cells causes synthetic lethality, as treated cells can neither remove proliferation-associated oxidative base damage by PARP-dependent base excision repair nor by the backup pathway HR at stalled DNA replication forks [6,7,8,9]. In this way, PARP inhibitors target HR-deficient tumor cells with comparatively few side effects in treated patients. **Olaparib**, the first-in-class PARP-inhibitor, was approved for the treatment of platinum-sensitive and BRCA-mutated types of ovarian cancer in the US and the EU in 2014, followed by numerous trials applying **Olaparib** or other PARP inhibitors as single agents or in combination therapies [10]. However, over the last years, tumor resistance mechanisms towards PARP inhibitor treatment were reported, such as reversion mutations in *BRCA1* and *BRCA2*, loss of *BRCA1* promoter hypermethylation, or overexpression of RAD51 [11]. In an attempt to overcome resistance, two series of hybrid ligands combining **Olaparib** with the histone-deacetylase (HDAC) inhibitor Vorinostat [12] and with the HSP90 inhibitor C0817 [13] have recently been developed. 

Familial breast and ovarian cancer, as well as to a lesser extent, also prostate and pancreatic cancer, have been linked with mutations in HR genes including but not limited to *BRCA1* and *BRCA2*, whose gene products act upstream of RAD51 in HR [14]. In exploiting the synthetic lethality principle beyond tumors with hereditary defects in the HR pathway, RAD51 as the key enzyme in HR, has become a novel therapeutic target in oncology [15] and several RAD51 inhibitors have already been reported [16]. In support of this concept, co-treatment of cells with a RAD51 inhibitor sensitizes breast cancer cells to single **Olaparib** treatment [17]. A dual PARP and RAD51 inhibitor might as well re-sensitize triple-negative breast cancer (TNBC) cells with hereditary and acquired defects in the BRCA1 pathway [17] after development of PARP inhibitor resistance reconstituting the HR machinery [18]. The molecular subtype of TNBC, accounting for approximately 10–15% of breast cancer with a classification based on absence of estrogen receptor (ER), progesterone receptor, and human epidermal growth factor receptor 2 (HER2) markers, is characterized by a more aggressive course of the disease and limited treatment options as compared to the hormone receptor-positive luminal and HER2-amplified breast cancer types [19].

**Figure 1:**
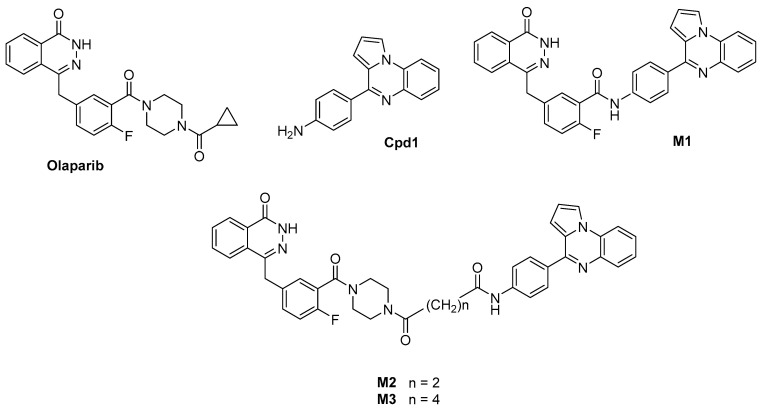
Structures of designed hybrid compounds **M1**–**M3** and of the parent drugs, PARP inhibitor **Olaparib**, and RAD51-inhibitor **Cpd1**. For further details regarding synthesis and analytical characterization, see Supplementary Material.

Here, we describe a novel class of anticancer drug conjugates **M1**–**M3** obtained by molecular hybridization of **Olaparib** and a recently reported blocker of RAD51-mediated D-loop formation (see Figure 1) [20]. This so-called Compound 1 (**Cpd1**) has been chosen, since it more specifically inhibits HR as compared with other RAD51 inhibitory agents, which also prevent formation of RAD51-ssDNA nucleoprotein filaments, and thereby inhibits both HR and replication fork stabilization [20]. Accordingly, the dual inhibitors will not interfere with colocalization of RAD51 and PARP1 at DNA damage sites such as stalled replication forks. The cell lines chosen for analyzing anti-survival effects of these drugs represented the clinically relevant subtypes of breast cancer, i.e., non-TNBC with luminal and HER2+ breast cancer cells as well as TNBC without and with *BRCA1* mutation.

## 2. Materials and Methods

### 2.1. Chemistry

The RAD51 inhibitor **Cpd1** has been prepared as previously reported [20]. The synthetic approach towards the drug conjugates **M1**-**M3** is reported in the Supplementary Material. Briefly, **M1** was prepared by amide formation between 5-[(3,4-Dihydro-4-oxo-1-phthalazinyl)methyl]-2-fluorobenzoic acid [21] and **Cpd1**. **M1** and **M2** were prepared by amide coupling of succinic acid and adipic acid monoethyl ester, respectively, with the amino group of **Cpd1** to give esters **2** and **3**, respectively, followed by ester hydrolysis and final amidation of the resulting acids with decyclopropanoyl olaparib [21]. Detailed experimental procedures including full analytical characterization by ^1^H-NMR, ^13^C-NMR, and LCMS are provided in the Supplementary Material. ^1^H- and ^13^C-NMR spectra, as well as the LC traces and ESI mass spectra, are shown in Supplementary Figures S1–S10. 

### 2.2. Cell Lines

MCF-7 (provided by American Type Culture Collection, ATCC, Manassas, Virginia, USA), MDA-MB-436 (provided by University Clinic Ulm, Ulm, Germany), MDA-MB-453 (provided by University Clinic Ulm, Ulm, Germany), MDA-MB-468 (provided by University Clinic Ulm, Ulm, Germany), and ZR75-1 (provided by Experimental Pharmacology and Oncology, Berlin-Buch, Berlin, Germany) were cultured in DMEM with L-glutamine (Gibco/ThermoFisher Scientific, Waltham, MA, USA), 10% FBS (Pan Biotech, Aidenbach, Germany), 1.2% L-glutamine (Gibco/ThermoFisher Scientific), 1.0% Penicillin-Streptomycin-Glutamine (100×) (Gibco/ThermoFisher Scientific), 1.0% MEM NEAA (non essential amino acid) (Gibco/ThermoFisher Scientific), 0.1% human recombinant insulin (Gibco/ThermoFisher Scientific), and 0.1% hEGF (Sigma-Aldrich/Merck, St. Louis, MO, USA) (Table 1) [22,23]. HCC-1937 cells (provided by ATCC) were cultured in RPMI 1640 medium with 15% FBS (Pan Biotech, Aidenbach, Germany) and 1% of Penicillin-Streptomycin-Glutamine (100X) (Gibco/ThermoFisher Scientific). Cells were cultured in a humid 5% CO_2_ incubator at 37 °C and all cell lines were negative for mycoplasma, verified by PCR.

### 2.3. Determination of Survival

The survival was determined by MTT assay (3-(4,5-dimethylthiazol-2-yl)-2,5-diphenyl tetrazolium bromide) (Sigma-Aldrich/Merck, St. Louis, MO, USA). First, the cells were seeded in 96-well plates at a density of 2 × 10^4^ cells per well. On day two, four, and seven, the cells were treated with **Olaparib** (TargetMol, Boston, MA, USA), **M1**, **M2**, **M3,** or **Cpd1** at 15 different treatment concentrations increasing up to 1024 µM in fresh culture medium each. On day nine, the MTT assay was performed. MTT was first dissolved in 1x PBS (Gibco/ThermoFisher Scientific) to a concentration of 5 mg/mL. Then, the 5 mg/mL MTT solution was diluted in OptiMEM (Gibco/ThermoFisher Scientific) and for HCC-1937 in RPMI without phenol red (Gibco/ThermoFisher Scientific) to a final concentration of 1 mg/mL. An amount of 100 µL of this solution were added to each well. After an incubation time of 2.5 h at 37 °C, the MTT solution was removed and the cells suspended in 200 µL 5% HCl/95% isopropanol (Sigma-Aldrich/Merck). The plates were shaken for 10 min and then the optical density measured at 570 nm with the Tecan Sunrise Photometer (Tecan, Crailsheim, Germany). The MTT experiments were conducted in duplicates and repeated twice each. 

### 2.4. γH2AX/RAD51 Immunofluorescence Microscopy

Cells were seeded on 4-well culture slides (Corning Inc., Corning, NY, USA) and incubated in 1 mL medium containing 10 µM **Olaparib** for 24 h. Slides were fixed with methanol (Sigma-Aldrich, St. Louis, MO, USA) at −20 °C for 10 min, washed three times in PBS, and blocked with 150 µL of 5% BSA (Sigma-Adrich, St-Louis, MO, USA) per chamber for 30 min at RT. Primary antibodies for RAD51 (anti-Rad51 rabbit H-92, sc-8349 from Santa Cruz Biotechnology, Dallas, TX, USA, 1:500 diluted in 5% BSA) and for γH2AX (anti-γH2AX mouse JBW 301, 05-636 from Merck Millipore, Burlington, MA, USA, 1:1000 in 5% BSA) were added and incubated at 4 °C overnight. Secondary anti-rabbit (Alexa Fluor 594 goat anti-rabbit IgG H+L, A11037 from Invitrogen/Thermo Fisher Scientific, Waltham, MA, USA, 1:500 diluted in 0.1% PBS-Tween) and anti-mouse antibodies (Alexa Fluor 488 goat anti-mouse IgG H+L, A11001 from Invitrogen/Thermo Fisher Scientific, 1:2000 diluted in 0.1% PBS-Tween) were added and incubated at 37 °C for 45 min protected from light. Slides were washed three times with 0.1% PBS-Tween and one time with PBS. Coverslips were mounted on slides using Vectashield mounting medium with DAPI and stored at 4 °C. The slides were subjected to high content imaging with a BZ-9000 microscope (Keyence, Neu-Isenburg, Germany), objective 100x/1.45 oil (Nikon), and automated analysis of immunostained foci in DAPI-stained nuclei with BZ-II Analyzer software (Keyence, Neu-Isenburg, Germany).

### 2.5. Western Blot Analysis

The cells were washed with ice-cold PBS, scraped in ice-cold PBS, and centrifuged. The pellet was suspended in 2–3 volumes of lysis buffer [50 mM Tris (Sigma-Aldrich/Merck, St. Louis, MO, USA), 150 mM NaCl (VWR international, Randor, PA, USA), 2 mM EGTA (Sigma-Aldrich/Merck, St. Louis, MO, USA), 2 mM EDTA (USB Chemicals, Thermo Fisher Scientific, Gujarat, India), 25 mM Sodium fluoride (Sigma-Aldrich/Merck, St. Louis, MO, USA), 25 mM Β-Glycerol phosphate (Merck, Darmstadt, Germany), 0.1 mM Sodium vanadate (Sigma-Aldrich/Merck, St. Louis, MO, USA), 0.2% Triton X-100 (Sigma-Aldrich/Merck, St. Louis, MO, USA), 0.3% Nonidet P40 (Fluka, München, Germany), 1 Protease inhibitor cocktail tablet (Sigma-Aldrich/Merck, St. Louis, MO, USA) per 8 mL]. A BCA-assay (Thermo Fisher Scientific, Waltham, Massachusetts, USA) was used to determine the protein concentration. We added 6x SDS sample buffer [350 mM Tris pH 6.8 (Sigma-Aldrich/Merck, St. Louis, MO, USA), 9.3% (*w/v*) DDT (Invitrogen/Thermo Fisher Scientific, Waltham, Massachusetts, USA), 10% (*w/v*) SDS (Merck, Darmstadt, Germany), 36% (*v/v*) Glycerol (Merck, Darmstadt, Germany), 0.6% (*w/v*) Bromophenol blue (Sigma-Aldrich/Merck, St. Louis, MO, USA), 15% 2-Mercaptoethanol (Sigma-Aldrich/Merck, St. Louis, MO, USA)] to the adjusted sample. Denaturation was done for 10 min at 95 °C. The SDS-Page with a separating gel containing 10% Acrylamide (Roth, Karlsruhe, Germany), was run at 80 V and the proteins were transferred for 50 min at 100 V onto a PVDF membrane (GE Healthcare, Chicago, IL, USA). Membranes were sequentially incubated overnight at 4 °C with the following primary antibodies diluted in 5% milk: anti-Rad51 rabbit H-92, sc-8349 from Santa Cruz Biotechnology, Dallas, TX, USA; mouse anti-α-Tubulin monoclonal antibody ab7291-100 from Abcam, Cambridge, UK, diluted 1:5000. Then, membranes were washed three times for 10 min with TBS-T [20 mM Tris, pH 7.6, 137 nM NaCl (VWR international, Randor, PA, USA), 0.1% Tween20 (Sigma-Aldrich/Merck, St. Louis, MO, USA)] followed by incubation for 1 h at RT with a secondary antibody anti-mouse or anti-rabbit (Rockland, PA, USA; 1:10.000). After three times of 10 min washing steps with TBS-T, the proteins were detected with Clarity^TM^ Western ECL Substrate (Bio-Rad Laboratories, Hercules, CA, USA). The proteins were visualized by ChemiDoc^TM^ MP Imaging System (Bio-Rad Laboratories, Hercules, CA, USA) and quantified with Image Lab 5.2.1 (Bio-Rad Laboratories, Hercules, CA, USA).

### 2.6. Statistics

Graphic presentation of cell viability curves was performed using GraphPadPrism versions 8 and 9 (GraphPad Software, San Diego, CA, USA). Statistical significance was determined using extra sum of-squares F-test, nonlin fit. Statistical significances (*p* values) of differences between mean IC_50_ values for unpaired, nonparametric data were first determined via Kruskal–Wallis test followed by a two-tailed Mann–Whitney-U test in case of statistical significance (*p* < 0.05). Statistics are shown in Supplementary Tables.

## 3. Results

Drug conjugates **M1**–**M3** have been designed by molecular hybridization of the PARP-inhibitor **Olaparib** and of the RAD51-inhibitor **Cpd1** [20] (Figure 1). In **M1**, the parental drugs are directly fused omitting the piperazine ring of **Olaparib** in a similar way as for the previously reported **Olaparib**-Vorinostat [12], and **Olaparib**-HSP90-inhibitor [13] anticancer hybrids. As for **M2** and **M3**, the two pharmacophores are linked by short -CO-(CH_2_)n-CO- spacers, n = 2 and 4, respectively. 

**Figure 2:**
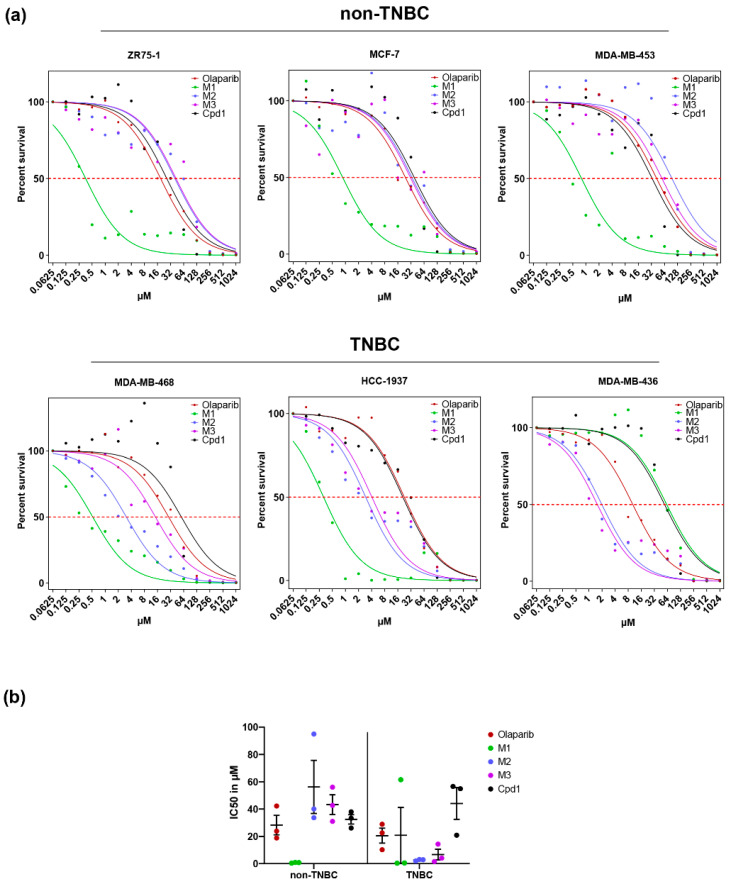
**Olaparib** and **Cpd1** hybrid compounds affect survival of non-TNBC and TNBC cells. Non-TNBC cells (ZR75-1, MCF-7 and MDA-MB-453) and TNBC cells (MDA-MB-468, HCC1937, and MDA-MB-436) were treated with different concentrations of **Olaparib**, **Cpd1**, **M1**, **M2**, and **M3**. (**a**) Non-TNBC cells showed high sensitivity to **M1** (green) with marked shifts of the survival curves to lower drug concentrations as compared to **Olaparib** (red) and **Cpd1** (black). All TNBC cells showed corresponding shifts of survival curves following treatment with **M2** (blue) or **M3** (magenta). However, except for the cell line MDA-MB-436, TNBC cells featured maximum sensitivities to **M1**. (**b**) Shown are the IC_50_ values calculated from survival curves in (**a**) for non-TNBC and TNBC cell lines. Each data point represents the mean of four values from two independent experiments.

The hybrid ligands were screened for their inhibitory effects on the viability of six different cancer cell lines, comprising two luminal (ZR75-1, MCF-7), one HER2+ (MDA-MB-453), and three TNBC lines without (MDA-MB-468) and with (HCC-1937, MDA-MD-436) pathogenic *BRCA1* mutations (Table 1) [22,23]. The dose-response survival curves are shown in Figure 2a, visualizing that the hybrid compound **M1** with **Olaparib** and **Cpd1,** directly connected by an amide linkage, kills five out of six cell lines included in our study with maximum efficiency, i.e., all lines with the exception of MDA-MB-436 cells. 

Comparison of the corresponding IC_50_ values (Table 2) for the drug conjugate **M1** reveals a 28- to 56-fold more pronounced inhibition of viability than achieved by the parental drug **Olaparib** and a 44- to 110-fold higher potency than parental **Cpd1** (*p* < 0.0001, see Statistics in Supplementary Tables). These data indicated a synergistic action of simultaneous PARP and RAD51 inhibition by **M1** in these five cell lines. 

As the only exception, MDA-MB-436 cells showed **M1** (IC_50_ = 61.5 µM) being 6-fold less potent compared with **Olaparib** (IC_50_ = 10.2 µM) and equipotent with **Cpd1** (IC_50_ = 54.9 µM). On the other hand, MDA-MB-436 cells were most sensitive to hybrid drugs with a spacer. Thus, IC_50_ values determined for **M2** and **M3** were 5- to 7-fold lower than for **Olaparib** and 28- to 35-fold lower than for **Cpd1** in MDA-MB-436 cells (*p* < 0.0001). 

When compared to the parental drugs, drug conjugates **M2** and **M3** with the two pharmacophores linked by short spacers did not sensitize non-TNBC cell lines (ZR75-1, MCF-7, MDA-MB-453) but the TNBC cell lines (MDA-MB-468, HCC-1937, MDA-MB-436) under investigation. As for MDA-MB-468 cells, a gradual increase of IC_50_ values was noticeable with increasing distance of the pharmacophores, whereby **M1** was 6-fold more potent than **M2** and **M2** 4-fold more potent than **M3** (*p* < 0.0001). As for HCC-1937 and MDA-MB-436, **M2** and **M3** were equipotent (*p* ≥ 0.1297) suggesting that the small difference in spacer length (two CH_2_-groups) between **M2** and **M3** had no major effect on cancer cell viability in these *BRCA1*-mutated cells.

Cell line group comparison of the average drug responses in Figure 2b highlights the striking potency of **M1** as compared to all other drugs in non-TNBC cells with >36-fold reduced mean IC_50_ compared with the values for parental drugs. This was true regardless of the p53 and HER2 status (Table 1). For the group of TNBC cells, **M2** with a two-carbon and **M3** with a four-carbon spacer between the parental drugs, showed >7- and >3-fold reduced mean IC_50_ values. These observations indicate that the mode-of-action for dual inhibitory drugs targeting PARP and RAD51 are subject to tight 3D constraints in non-TNBC cell lines, while in TNBC cell lines a larger degree of flexibility exists. 

Of note, both control drugs **Olaparib** and **Cpd1** similarly reduced cell survival of all six cancer cell lines with IC_50_ values ranging from 10–57 µM. Thus, though MDA-MB-436 showed the highest sensitivity to **Olaparib** (IC_50_ = 10.21 µM), as would be predicted for *BRCA1*-mutated cells [8], the **Olaparib** response of HCC-1937 cells was indistinguishable from the one in the luminal cell lines ZR75-1 and MCF7. In support, Pierce and colleagues [24] determined similar IC_50_-values for MCF-7 and HCC-1937 cells after **Olaparib** treatments and found no statistically significant difference between IC_50_ values from seven non-TNBC and 7 TNBC cell lines in both MTT as well as colony formation assays. 

**Figure 3:**
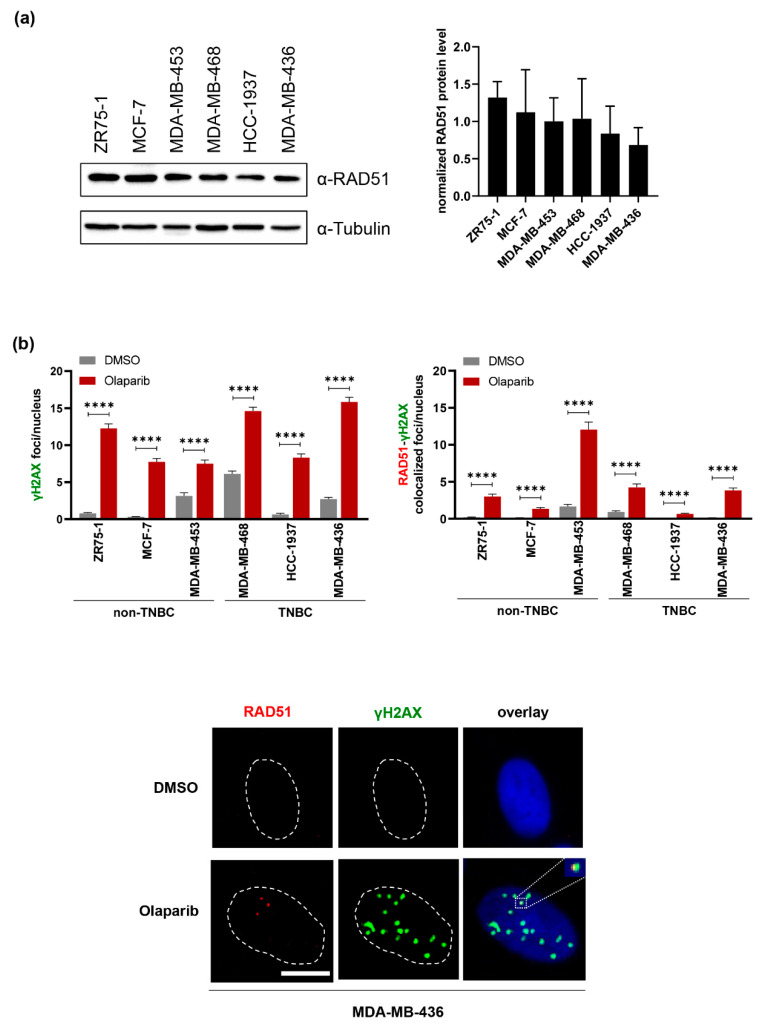
Total RAD51 levels, nuclear γH2AX foci, and colocalization with RAD51 foci. Non-TNBC cell lines (ZR75-1, MCF-7, MDA-MB-453) and TNBC cell lines (MDA-MB-468, HCC-1937, MDA-MB-436) were left untreated for Western Blotting or treated with DMSO and **Olaparib** for microscopic analysis of γH2AX (Ser139) and RAD51. (**a**) Total protein levels of RAD51 were analyzed after Western Blotting by quantification of band intensities and normalization to the loading control α-Tubulin (left, representative blot; right, graphic presentation of mean values +SD from two experiments). (**b**) Immunofluorescence microscopy was performed after DMSO and **Olaparib** (10 µM) treatment for 24 h and staining for γH2AX and RAD51. Focal accumulations of γH2AX and γH2AX foci colocalizing with RAD51 were counted per nucleus in two individual experiments and mean values +SEM displayed graphically on top (γH2AX: 310-506 nuclei; colocalization of γH2AX-RAD51: 310-529 nuclei; **** *p* < 0.0001). Exemplary microscopic images are presented for cell line MDA-MB-436 in the bottom. White dashed lines encircle DAPI-stained nuclei and the inset displays the highlighted region at two-fold magnification. Scale bar indicates 10 µM.

To provide additional information on the RAD51 status in these cell lines, we produced Western Blotting and immunofluorescence microscopic data of RAD51 foci formation at **Olaparib**-induced DNA damage sites marked by γH2AX (Figure 3). These results indicate that the RAD51 protein level was similar in all cell lines, even though a trend of reduced RAD51 expression was noticed in the *BRCA1*-mutated HCC1937 and MDA-MB-436 cells (Figure 3a). Interestingly, however, **Olaparib**-induced RAD51 foci at γH2AX-marked DNA damage sites in MDA-MB-436 cells were in the range of RAD51 foci numbers in non-TNBC or MDA-MB-468 TNBC cells but close to the detection limit in HCC-1937 cells despite γH2AX foci numbers in the normal range (Figure 3b). These data suggest compromised nucleoprotein filament assembly in HCC-1937 cells but not MDA-MB-436 cells, i.e., adaptation in the latter ones.

Finally, we also compared single versus combined **Olaparib** and **Cpd1** treatments in ZR75-1, MCF-7, MDA-MB-468, and MDA-436 cells (Supplementary Figure S11). Interestingly, in none of these cell lines did combined treatment reduce the IC_50_ values to the same extent as was observed for dual inhibitor treatment. Thus, no significantly different IC_50_ values (e.g., for **Olaparib** versus **Olaparib** plus **Cpd1** in MDA-MB-468 and MDA-MB-436) or at most 4.7-fold different IC_50_ values (e.g., for **Cpd1** versus **Olaparib** plus **Cpd1** in ZR75-1) were observed for combined versus single treatments. For comparison, ≤110-fold differences were achieved with dual inhibitor treatments. From this, we conclude that the dual inhibitor synergistically enhances cell killing, an effect that is not reached by combined **Olaparib** and **Cpd1** treatments, at least not to the same extent.

Altogether these findings indicate that neither PARP nor RAD51 inhibition alone nor combined drug treatment can discriminate between these groups of cell lines with in part differing RAD51 status. However, dual inhibition can break pre-existing PARP inhibitor resistance mechanisms bypassing BRCA1 dysfunction in HR and replication fork protection such as previously described for HCC-1937 [18].

## 4. Discussion

Since their first approval in 2014, PARP inhibitors entered multiple treatment regimens to combat hereditary and sporadic tumor types including but not limited to *BRCA1*- and *BRCA2*-mutated malignancies [25,26]. While side-effects of PARP inhibitory drugs are comparatively minor, acquisition of drug resistance has become the major challenge [27]. Given that PARP inhibitor resistance mechanisms can escape synthetic lethality by reconstituting the HR machinery in the tumor cells, several clinical trials combining the PARP inhibitor with another DNA repair inhibitory drug have been launched [25,26]. Here, we chose an alternative strategy by designing dual inhibitory drugs targeting both PARP and the key HR molecule RAD51. Thereby, we aimed at a spatial coupling of the two mechanisms underlying synthetic lethality and an increase of the local concentration of both inhibitors at the site-of-action, potentially reducing the pharmacologically necessary dose. Indeed, we found that in all cell lines, at least one of the dual inhibitory drugs **M1**, **M2**, or **M3** reduced the IC_50_ by one to two orders of magnitude compared to the parental drugs. Most interestingly, the drug conjugate **M2** with a -CO-(CH_2_)_2_-CO- spacer between the parental drug moieties discriminated between non-TNBC and TNBC cell lines with sensitivities differing by >20-fold. **M3,** with a two-carbons-longer -CO-(CH_2_)_4_-CO- spacer, also clearly separated these cell line sets but to a lesser extent than **M2**.

Such a large impact of spacing argues for a role of PARP1 and RAD51 in a highly coordinated process in close proximity, very likely within the same nucleoprotein complex. Of note, recent work revealed that both PARP1 and RAD51 are not only key enzymes in base excision repair and HR, respectively [6,9], but are as well engaged in the controlled reactivation of stalled replication forks. Thus, meta-stable RAD51 nucleoprotein filaments aid fork reversal catalyzed by different translocases. Thereafter, stable RAD51 filaments and PARP1 protect reversed forks from unscheduled nucleolytic processing and remodeling by RECQ1, respectively [28,29]. **Cpd1** is the only RAD51 inhibitor that specifically blocks D-loop formation during HR but not ssDNA binding, filament formation, or fork reversal by RAD51 in the concentration range up to the IC_50_ values determined for breast cancer cell lines in our study [20]. Therefore, **Olaparib**-**Cpd1** hybrid drugs should permit RAD51 loading and colocalization with PARP1 at stalled replication forks [30]. Of note, **Olaparib** treatment is known to trap PARP1 by allosteric effects on DNA, which correlates with cell killing and which was suggested to guide the development of improved drugs [31]. Therefore, synergy observed in our study with hybrid drugs could be caused by locking PARP1 on reversed forks adjacent to RAD51 filaments, which will lead to fork collapse, i.e., DNA double-strand breaks (DSBs) which, however, can no longer be repaired by HR.

The HR factors BRCA1 and BRCA2 have also been reported to protect forks, namely from nucleolytic attack through stabilization of RAD51 filaments [28]. Therefore, depletion of BRCA1, as frequently caused in TNBC cell lines by genetic or epigenetic mechanisms [17], will increase the plasticity of the fork. Such a scenario can explain why the BRCA1-positive non-TNBC cells were most sensitive to the spacer-free compound **M1**, suggesting close neighborhood between PARP1 and RAD51. Compatible with loosened spatial constraints, TNBC cell lines showed broader and more heterogeneous responses, with significant cell killing by **M2** and **M3** and in part also by **M1**. While the *BRCA1*-mutated line HCC-1937 showed a gradual decrease of cell killing with the length of the spacer between the parental drugs, BRCA1-depleted MDA-MB-436 cells exhibited the best response to **M2** and **M3**. In MDA-MB-436 cells, the IC_50_ values for **M1** and **Cpd1** were both even higher than for **Olaparib**, suggesting that **M1** cannot simultaneously target PARP1 and RAD51. At this point, we can only speculate about potential changes in MDA-MB-436 cells impacting on interdomain communication between the C-terminal **Olaparib**- and N-terminal DNA-binding domains of PARP1 [31] or on the properties of residual RAD51 nucleoprotein filaments abrogating such dual interactions of **M1**. Ultimately, 3D structural analyses of the hybrid drugs bound to their targets are warranted to answer these mechanistically interesting questions.

Regarding candidate signaling pathways to explain these changes in MDA-MB-436 cells, we notice that PTEN, known to upregulate RAD51 [32], is deleted in MDA-MB-468 and HCC-1937 but not in MDA-MB-436 cells [19,33]. Here, we did not find elevated RAD51 expression in MDA-MB-436 cells. MDA-MB-436 cells were also reported to rapidly acquire *BRCA1* reversion mutations partially reconstituting HR [34]. Indeed, in a previous study, we measured ≥10-fold higher HR frequencies in MDA-MB-436 compared with other breast cancer cell lines including MCF7, MDA-MB-453, and MDA-MB-468 [23]. In agreement with HR proficiency, here we observed RAD51 nucleoprotein filament formation at sites of **Olaparib**-induced DNA damage. Therefore, it is conceivable that MDA-MB-436 cells quickly adapted to BRCA1 loss by rewiring their RAD51 status similar to the pre-existing resistance mechanisms described for HCC-1937 cells [18]. Further resistance mechanisms, such as reduced DNA end protection by 53BP1 seen in all the TNBC cell lines investigated in our study [35], may explain why **Olaparib** sensitivities were in general similar in the TNBC as compared with non-TNBC cell lines.

## 5. Conclusions

In conclusion, **Olaparib**-RAD51 inhibitor conjugates have the potential to break resistance mechanisms to **Olaparib** treatment in TNBC cells and sensitize breast cancer cells regardless of BRCAness. While close proximity of the parental drugs in the hybrid is critical for their efficacy in non-TNBC cells, a separation of both pharmacophores by-CO-(CH_2_)_2_-CO- and CO-(CH_2_)_4_-CO spacers leads to TNBC cell killing. Specific targeting of PARP1 at sites of DNA repair and replication may be desirable when treating oncological diseases to avoid non-specific effects related to other PARP1 functions, such as a regulator of necrosis and inflammation in a sex-specific manner [6,36].

## Figures and Tables

**Table 1:** Characteristics of breast cancer cell lines.

Cell Line	Tumor Source	Molecular Subtype	ER Status	BRCA1 Status	p53 Status
ZR75-1	Metastatic breast cancer	luminal	+	wt	wt
MCF-7	Metastatic breast cancer	luminal	+	wt	wt
MDA-MB-453	Metastatic breast cancer	HER2+	-	wt	p.? (c.1101_1130del)
MDA-MB-468	Metastatic breast cancer	TN	-	wt	p.R273H
HCC-1937	Primary ductal carcinoma	TN	-	p.Q1756fs*74	p.R306*
MDA-MB-436	Metastatic breast cancer	TN	-	p.? (c.5396+1G>A)	p.? (c.612_613ins7)

Information was taken from IARC TP53 Database and [22,23]; ER, estrogen receptor; HER2, human epidermal growth factor receptor 2; TN, triple-negative; wt, wild-type. “?” means unknown and “*” means termination codon.

**Table 2:** Sensitivity of breast cancer cell lines to single and dual inhibitory drugs.

Cell Line	IC_50_ [µM]				
	Olaparib	M1	M2	M3	Cpd1
ZR75-1	18.83	0.3553	40.08	42.70	26.12
MCF-7	23.82	0.8632	33.64	30.99	37.95
MDA-MB-453	42.26	0.7947	94.97	56.01	33.31
MDA-MB-468	28.87	0.5172	3.064	14.33	56.68
HCC-1937	22.54	0.3147	2.924	4.056	20.84
MDA-MB-436	10.21	61.51	1.982	1.564	54.90

IC_50_, inhibitory concentration 50.

## Data Availability

The data presented in this study are available on request from the corresponding authors.

## Supplementary Materials

The following are available online at https://www.mdpi.com/article/10.3390/biom11070981/s1. Experimental procedures for the synthesis of compounds **M1**-**M3**. Supplementary Figures S1: ^1^H and ^13^C-NMR-spectra of **Cpd1;** S2: ^1^H and ^13^C-NMR-spectra of **M1**; S3: ESI-LCMS data of **M1**; S4: ^1^H and ^13^C-NMR-spectra of compound **2**, S5: ESI-LCMS data of compound **2**; S6: ^1^H and ^13^C-NMR-spectra of compound **3**; S7: ^1^H and ^13^C-NMR-spectra of **M2**; S8: ESI-LCMS data of **M2**; S9: ^1^H and ^13^C-NMR-spectra of **M3**; S10: ESI-LCMS data of **M3**; S11: Single versus combined addition of **Olaparib** and **Cpd1.** Supplementary Tables ad Figure S2A and Table S2: Statistical comparisons for single and dual inhibitory compound treatments; Supplementary Tables ad Supplementary Figure S11: Statistical comparison for single and combined drug treatments.
