# Supplementary material for: Dual PARP and RAD51 Inhibitory Drug Conjugates Show Synergistic and Selective Effects on Breast Cancer Cells
Source: Biomolecules. 2021 Jul 3;11(7):981. doi: 10.3390/biom11070981 (PMC8301877; doi:10.3390/biom11070981)
Supplement: Supplementary file 1 [file biomolecules-11-00981-s001.zip › biomolecules-1247289-supplementary.pdf]

Supplementary Material

Dual PARP1 and RAD51 Inhibitory Drug Conjugates Show Synergistic and Selective Effects on Breast Cancer Cells

Matthews M. Malka[1#], Julia Eberle[2#], Kathrin Niedermayer[2], Darius P. Zlotos[1*§] and Lisa Wiesmüller[2*§]

[1]Department of Pharmaceutical Chemistry, The German University in Cairo, New Cairo City, Main Entrance of Al Tagamoa Al Khames, 11835 Cairo, Egypt; darius.zlotos@guc.edu.eg
[2]Department of Obstetrics and Gynecology, Ulm University, Prittwitzstrasse 43, 89075 Ulm, Germany; lisa.wiesmueller@uni-ulm.de

Experimental Procedures for the Synthesis of compounds M1-M3

Reaction Schemes	page 2
General Experimental Procedures	page 3
Synthesis of **M1**	page 4
Synthesis of Esters **2** and **3**	page 5
Synthesis of Acids **4** and **5**	page 6
Synthesis of Drug Conjugates **M2** and **M3**	pages 7-8

Reaction Schemes.

M1 was prepared by amide formation between 5-[(3,4-Dihydro-4-oxo-1-phthalazinyl)methyl]-2-fluorobenzoic acid and **Cpd1**. **M1** and **M2** were prepared by amide coupling of succinic acid and adipic acid monoethyl ester, respectively, with the amino group of **Cpd1** to give esters **2** and **3**, respectively, followed by ester hydrolysis and final amidation of the resulting acids **4** and **5** with decyclopropanoyl **Olaparib**.

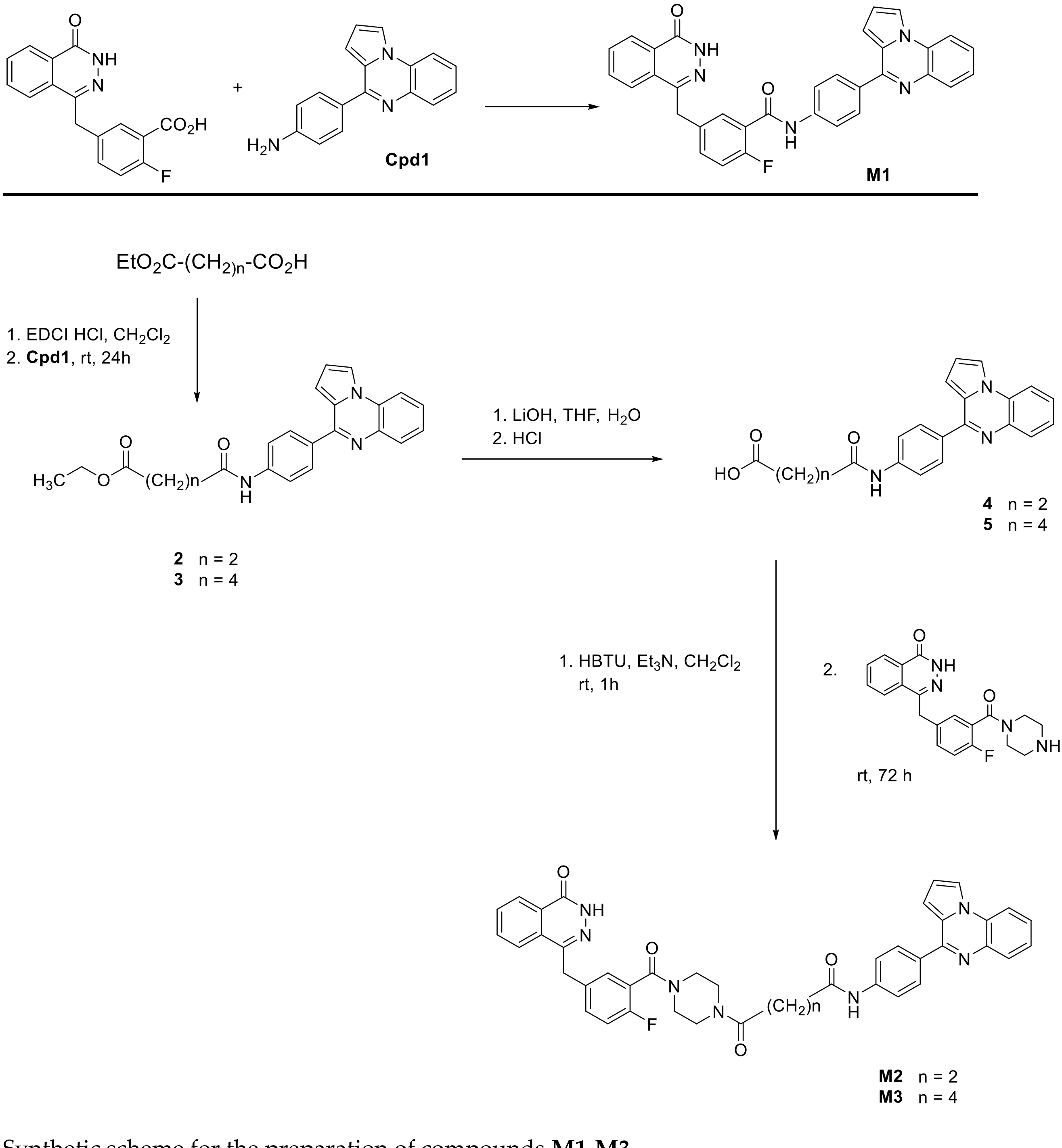

Synthetic scheme for the preparation of compounds **M1-M3**

General Experimental Procedures.

Melting points were determined using a capillary melting point apparatus (Gallenkamp, Sanyo) and are uncorrected. A Bruker AV-400 spectrometer was used to obtain ^{1}H NMR and ^{13}C NMR spectra, respectively. ^{1}H NMR chemical shifts are referred to $CHCl_3$ (7.26 ppm) and DMSO-d_6 (2.50 ppm). ^{13}C NMR chemical shifts are referred to $CDCl_3$ (77.00 ppm) and DMSO-d_6 (39.52 ppm). All reactions were carried out under an argon atmosphere. Column chromatography was carried out on silica gel 60 (0.063–0.200 mm) obtained from Merck (Darmstadt, Germany). TLC analysis was performed on commercial silica gel 60 F254 aluminum sheets (Macherey-Nagel, Düren. Germany). Analytical HPLC for compound **M1** was performed using Waters ACQUITY XevoTQD UPLC-ESI-MS system, which consisted of an ACQUITY UPLC H-Class system, Xevo™ TQD triple-quadrupole tandem mass spectrometer and an electrospray ionization (ESI) interface (Waters Corp., Milford, MA, USA). Acquity BHE C18 100 mm × 2.1 mm column (particle size, 1.7 μm) was used to separate analytes (Waters, Ireland). The solvent system was composed of water containing 0.1% TFA in acetonitrile. Analytical HPLC for compounds **M2**, and **M3** was performed on a system from Shimadzu Products equipped with a DGU-20A3R controller, LC20AB liquid chromatograph, and an SPD-20A UV/Vis detector. Stationary phase was a Phenomenex Synergi 4U fusion-RP (150 × 4.6 mm) column. As mobile phase, a gradient of MeOH/water was used (solvent A, water with 0.1% formic acid; solvent B, MeOH with 0.1% formic acid; gradient: solvent B from 0% to 90% in 13 min, then at 90% for 5 min, from 90% to 5% in 1 min, and then 5% for 4 min, flow rate of 1 mL, UV-detection was measured at 254 nm). 2-Fluoro-5-[(4-oxo-3,4-dihydrophthalazin-1yl)methyl]benzoic acid and 4-{4-Fluoro-3-[(piperazine-1-carbonyl)benzyl]} phtalazin-1(2H)-one have been prepared according to Zmuda et al. J. Med. Chem. (2015) 58:8683. **Cpd1** has been prepared according to Lv et al. J. Med. Chem. (2016) 59: 4511.

Synthesis of M1.

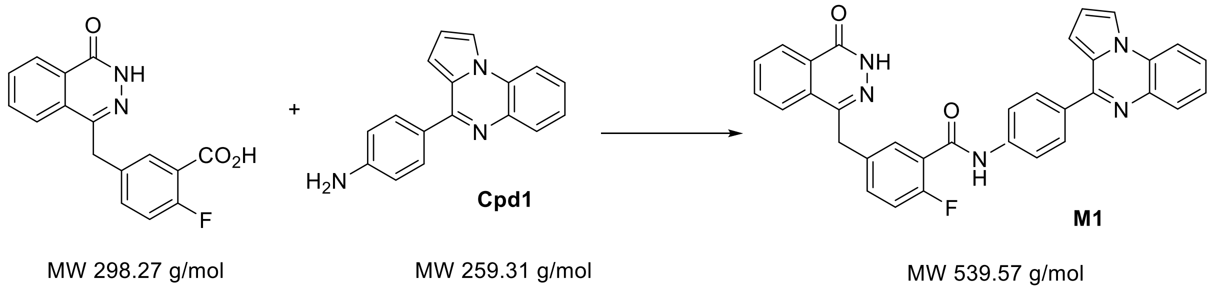

2-Fluoro-5-[(4-oxo-3,4-dihydrophthalazin-1yl)methyl]-*N*-[(4-pyrrolo[1,2-*a*]quinoxaline-4-yl)phenyl]benzamide (**M1**)

2-Fluoro-5-[(4-oxo-3,4-dihydrophthalazin-1yl)methyl]benzoic acid (298 mg, 1 mmol) was dissolved in dry DMF (30 mL). Triethylamine (0.2 mL, 1.6 mmol) and HBTU (420mg, 1.1 mmol) were added and the reaction mixture was stirred at room temperature for 1 hour. Compound **1** (259 mg, 1 mmol) was added and stirring was continued for 72 hours. The mixture was diluted with water (40ml), stirred at 50^0C for 2 hours and cooled to 0^0C. The precipitated solid crude product was collected by vacuum filtration and purified by column chromatography using EtOAc as eluent to yield **M1** (200 mg, 36%) as a yellow solid. ^{1}H NMR (400 MHz, $CDCl_3$) δ 8.30 (1H, dd, *J* = 7.1, 1.5 Hz), 7.97-7.95 (1H, m), 7.91-7.83 (4H, m), 7.81 (1H, d, *J* = 8.2 Hz), 7.76 (2H, m), 7.71-7.63 (5H, m), 7.43 (1H, m), 7.36-7.28 (2H, m), 7.03 (1H, m), 6.92 (1H, m), 6.82 (1H, dd, *J* = 4.0, 2.7 Hz), 4.26 (2H, s); ^{13}C NMR (101 MHz, mixture of $CDCl_3$ and DMSO-*d6*), δ 176.7 (C=O), 160.7 (C=O), 145.8 (C), 153.7 (C), 145.3, (C), 139.2 (C), 133.6 (CH), 131.5 (CH), 131.4 (CH, d, *J* = 8.1 Hz), 129.3 (CH), 129.27 (C), 129.0 (C), 127.9 (CH), 127.5 (CH), 126.7 (CH), 125.8 (C), 125.3 (CH), 124.9 (C), 121.5 (CH), 120.1 (CH), 116.5 (CH, d, *J* = 24.8 Hz), 115.1 (C), 114.8 (CH), 114.2 (CH), 113.6 (C), 110.0 (C), 109.3 (C), 37.5 (CH_2), (three quaternary carbon atoms in the fluorinated benzene ring supposedly appearing as dubletts due to C-F couplings are not detectable); R_f = 0.55 (EtOAc); HPLC-purity 91.8 %; tR 8.75 min; ESIMS *m*/*z* 540.28 $[M+H]^+$, 270.84 $[M+2H]^{2+}$.

Synthesis of Esters 2 and 3.

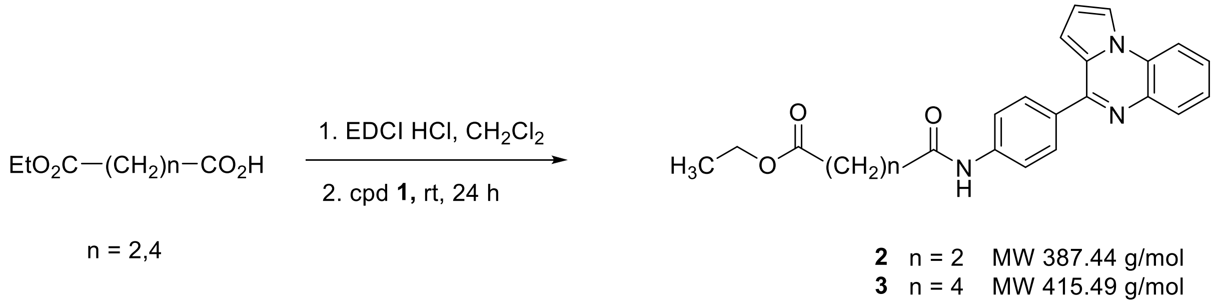

The respective acid (0.692 mmol) was dissolved in in dry dichloromethane (20 mL) and the solution was cooled under ice-water bath. EDCI HCl (133 mg, 0.692 mmol) was added and the reaction mixture was stirred for 15 min. Compound **1** (180 mg, 0.694 mmol) was added, the cooling bath was removed, and stirring was continued for 24 h. The solvent was removed in vacuo and the residue was purified by column chromatography using chloroform/methanol/ 25% NH_3 100:10:1 as eluent to give the respective ester **2** or **3**.

Ethyl 4-oxo-4-{[(4-(pyrrolo[1,2-a]quinoxaline-4-yl)phenyl]amino}butanoate (**2**)
Compound **2** (121 mg, 45%) was obtained from monoethyl succinate EtO_2C-$(CH_2)_2$-CO_2H (101 mg, 0.692 mmol) as yellow solid. ^1H NMR (400 MHz, $CDCl_3$) δ 8.61 (1H, s), 7.96 (1H, dd, *J* = 8.0, 1.4 Hz), 7.94 (1H, d, *J* = 8.5 Hz), 7.90 (1H, d, *J* = 1.7 Hz), 7.79 (1H, d, *J* = 8.0 Hz), 7.71 (1H, d, *J* = 8.5 Hz), 7.44 (1H, m), 7.38 (1H, td, *J* = 7.8, 1.0 Hz), 4.16 (2H, q, *J* = 7.1 Hz), 2.74 (2H, dd, *J* = 10.0, 3.9 Hz), 2.67 (2H, dd, *J* = 10.0, 3.9 Hz), 1.25 (3H, t, *J* = 7.1 Hz). ^{13}C NMR (101 MHz, $CDCl_3$) δ 173.1 (C=O), 170.1 (C=O), 153.5 (C), 139.6 (C), 136.0 (C), 133.8 (C), 129.7 (CH), 129.3 (CH), 127.2 (CH), 126.9 (C), 125.10 (CH), 125.05 (C), 119.4 (CH), 114.5 (CH), 113.9 (CH), 113.5 (CH), 108.6 (CH), 60.8 (CH_2), 31.8 (CH_2), 29.3 (CH_2), 14.0 (CH_3); R_f = 0.20 ($CHCl_3$, MeOH, 25% NH_3/ 100:10:1); HPLC-purity 100.0 %; tR 9.40 min; ESIMS *m/z* 416.05 $[M+H]^+$.

Ethyl 6-oxo-6-{[(4-(pyrrolo[1,2-a]quinoxaline-4-yl)phenyl]amino}hexanoate (**3**)
Compound **3** (118 mg, 41%) was obtained from adipic acid monoethyl ester EtO_2C-$(CH_2)_4$-CO_2H (121 mg, 0.692 mmol) as yellow solid. ^1H NMR (400 MHz, $CDCl_3$) δ 8.47 (1H, s), 7.99 7.91 (2H, m), 7.81 (1H, d, *J* = 8.0 Hz), 7.74 (1H, d, *J* = 8.4 Hz), 7.45 (1H, m), 7.39 (1H, t, *J* = 7.5 Hz), 4.12 (2H, q, *J* = 7.1 Hz), 2.32 (2H, t, *J* = 7.1 Hz), 1.79-1.62 (4H, m), 1.25 (3H, t, *J* = 7.1 Hz); HPLC-purity 100 %; tR 9.398 min; ESIMS *m/z* 416.05 $[M+H]^+$, ^{13}C NMR (101 MHz, $CDCl_3$) δ 173.6 (C=O), 171.3 (C=O), 153.6 (C), 139.7 (C), 136.0 (C), 133.7 (C), 129.7 (CH), 129.3 (CH), 127.3 (CH), 127.0 (C), 125.2 (CH), 125.1 (C), 119.5 (CH), 114.6 (CH), 114.0, 113.6 (CH), 108.8 (CH), 60.3 (CH_2), 36.9 (CH_2), 33.8 (CH_2), 24.8 (CH_2), 24.2 (CH_2), 14.1 (CH_3); R_f = 0.23 ($CHCl_3$, MeOH, 25% NH_3/ 100:10:1).

Synthesis of Acids 4 and 5.

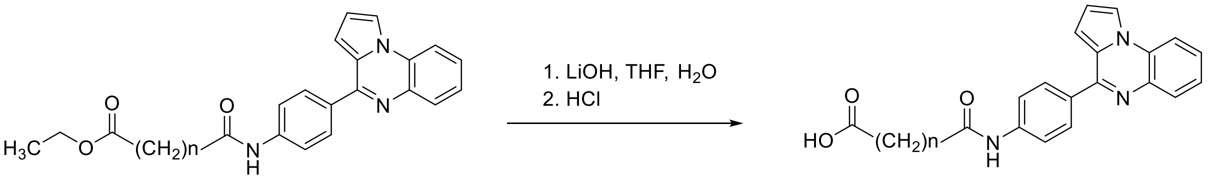

2 n = 2
3 n = 4

4 n = 2 MW 359.39 g/mol
5 n = 4 MW 387.44 g/mol

2M aqueous LiOH solution (10 mL) was added to a stirred solution of the respective ester (**2** or **3)** in THF (10 mL). After stirring at room temperature for 24 h, THF was evaporated under reduced pressure. The aqueous residue was diluted with water (20 mL) and acidified using 2M HCl. The reaction mixture was extracted with dichloromethane (5 x 10 mL). The combined organic phases were washed with water, dried over Na_2SO_4 and concentrated under reduced pressure to give the respective crude acid **4** or **5** that was used for the next step without further purification.

4-Oxo-4-{[(4-(pyrrolo[1,2-a]quinoxaline-4-yl)phenyl]amino}butanoic acid (**4**). Compound **4** (230 mg, 64%) was obtained from **2** (387 mg, 1 mmol) as yellow solid¸ R_f = 0.02 ($CHCl_3$, MeOH, 25% NH_3/ 100:10:1).
6-oxo-6-{[(4-(pyrrolo[1,2-a]quinoxaline-4-yl)phenyl]amino}hexanoic acid (**5**). Compound **5** (260 mg, 67%) was obtained from **3** (415 mg, 1 mmol) as yellow solid, R_f = 0.02 ($CHCl_3$, MeOH, 25% NH_3/ 100:10:1).

Synthesis of Drug Conjugates M2 and M3.

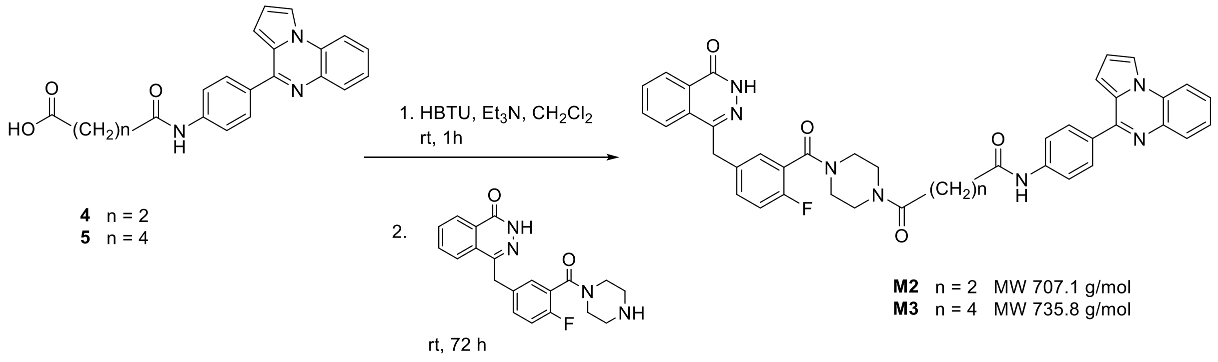

The crude acid was dissolved in dry dichloromethane (30 mL), NEt_3 (0.09 mL, 0.6 mmol) and HBTU (180 mg, 0.4 mmol) were added and the reaction mixture was stirred at room temperature for 1 h. 4-{4-Fluoro-3-[(piperazine-1-carbonyl)benzyl]}phtalazin-1(2H)-one (135 mg, 0.37 mmol) was added at 50^{0}C and stirring was continued at room temp. for 72 h. The solvent was removed under reduced pressure and the residue was purified by column chromatography using chloroform/ methanol/ 25% NH_3 100:10:1 as eluent to give the drug conjugate **M2** or **M3**.

4-{4-[2-fluoro-5-((4-oxo-3,4-dihydrophthaazin-1-yl)methyl)benzoyl]piperazin-1-yl}-4-oxo-*N*-[4-(pyrrolo[1,2-a]quinoxaline-4-yl)phenyl]butanamide (**M2**).
Compound **M2** (104 mg, 40%) was obtained from the acid **4** (132 mg, 0.37 mmol) as white foam. ^{1}H NMR (400 MHz, $CDCl_3$) δ 10.94-10.87 (1H, m), 8.75 (1H, d, *J* = 5.2 Hz), 8.49-8.44 (1H, m), 8.00 (1H, d, *J* = 7.8 Hz), 7.97-7.93 (3H, m), 7.85 (1H, d, *J* = 7.4 Hz), 7.80-7.66 (5H, m), 7.49 (1H, td, *J* = 7.8, 1.4 Hz), 7.46-7.39 (1H, m), 7.38-7.27 (2H, m), 7.03 (1H, t, *J* = 8.7 Hz), 6.96 (1H, dd, *J* = 4.0, 1.0 Hz), 6.87-6.82 (1H, m), 4.26 (2H, s), 3.85-3.71 (3H, m), 3.65-3.47 (3H, m), 3.51-3.45 (2H, m), 2.85-2.75 (4H, m); ^{13}C NMR (101 MHz, $CDCl_3$) δ 170.9 (C=O), 170.7 (C=O), 160.4 (C=O), 153.6 (C), 145.5 (C), 145.3, (C), 136.2 (C), 133.6 (CH), 131.6 (CH), 131.8 (CH, d, *J* = 8.1 Hz), 130.0 (CH), 129.5 (C), 129.4 (CH), 128.3 (C), 127.3 (CH), 127.2 (C), 127.1 (CH), 125.2 (CH), 124.9 (CH), 119.3 (CH), 116.2 (CH, d, *J* = 25 Hz), 114.6 (CH), 114.0 (CH), 113.6 (CH), 108.6 (CH), 46.7 (CH_2), 45.0 (CH_2), 42.0 (CH_2), 37.6 (CH_2),32.6 (CH_2), 28.7 (CH_2) (three quaternary carbon atoms in the fluorinated benzene ring supposedly appearing as dubletts due to C-F couplings are not detectable); R_f = 0.11 ($CHCl_3$, MeOH, 25% NH_3/ 100:10:1); HPLC-purity 98.1 %; tR 8.46 min; ESIMS *m*/*z* 708.15 $[M+H]^+$.

6-{4-[2-fluoro-5-((4-oxo-3,4-dihydrophthaazin-1-yl)methyl)benzoyl]piperazin-1-yl}-6-oxo-*N*-[4-(pyrrolo[1,2-a]quinoxaline-4-yl)phenyl]hexanamide (**M3**).
Compound **M3** (114 mg, 42%) was obtained from compound **5** (143 mg, 0.37 mmol) as yellow solid. ^{1}H NMR (400 MHz, $CDCl_3$) δ 11.13-11.03 (1H, m), 8.88-8.83 (1H, m), 8.46-8.43 (1H, m), 8.01 (1H, dd, *J* = 7.9, 1.4 Hz), 7.98-7.95 (3H, m), 7.85 (1H, d, *J* = 7.4 Hz), 7.82-7.63 (5H, m), 7.51-7.46 (1H, m), 7.46-7.40 (1H, m), 7.33-7.28 (2H, m), 7.02 (1H, t, *J* = 8.8 Hz), 6.98 (1H, d, *J* = 3.8 Hz), 6.87 (1H, dd, *J* = 3.8, 2.9 Hz), 4.25 (2H, s), 3.85-3.71 (3H, m), 3.62-3.51 (3H, m), 3.41-3.23

(2H, m), 2.46-2.32 (4H, m), 1.80-1.70 (4H, m); ^{13}C NMR (101 MHz, $CDCl_3$) δ 171.7 (C=O), 171.5 (C=O), 165.3 (C), 160.5 (C=O), 157.0 (C, d, *J* = 248 Hz), 153.7 (C), 145.4 (C), 140.0, (C), 136.2 (C), 134.5 (C, d, *J* = 3 Hz), 133.6 (CH), 131.8 (CH, d, *J* = 8.1 Hz), 131.6 (CH), 130.0 (CH), 129.5 (C), 130.0 (CH), 129. 5 (C), 129.3 (CH), 128.3 (C), 127.3 (CH), 127.10 (C), 127.06 (CH), 125.2 (CH), 124.9 (CH), 123.5 (C, d, *J* = 18 Hz), 119.5 (CH), 116.2 (CH, d, *J* = 25 Hz), 114.6 (CH), 114.0 (CH), 113.6 (CH), 108.7 (CH), 46.7 (CH_2), 45.0 (CH_2), 42.1 (CH_2), 41.8 (CH_2), 37.6 (CH_2), 37.1 (CH_2), 32.6 (CH_2), 24.9 (CH_2), 24.2 (CH_2); R_f = 0.11 ($CHCl_3$, MeOH, 25% NH_3/ 100:10:1); HPLC-purity 98.7 %; tR 9.281 min; ESIMS *m*/*z* 736.10 $[M+H]^+$.

Supplementary Material

Dual PARP1 and RAD51 Inhibitory Drug Conjugates Show Synergistic and Selective Effects on Breast Cancer Cells

Matthews M. Malka[1#], Julia Eberle[2#], Kathrin Niedermayer[2], Darius P. Zlotos[1*§] and Lisa Wiesmüller[2*§]

[1]Department of Pharmaceutical Chemistry, The German University in Cairo, New Cairo City, Main Entrance of Al Tagamoa Al Khames, 11835 Cairo, Egypt; darius.zlotos@guc.edu.eg
[2]Department of Obstetrics and Gynecology, Ulm University, Prittwitzstrasse 43, 89075 Ulm, Germany; lisa.wiesmueller@uni-ulm.de

Supplementary Figures S1-S11

Fig. S1	^{1}H and ^{13}C-NMR-spectra of **Cpd1**	page 2
Fig. S2	^{1}H and ^{13}C-NMR-spectra of **M1**	page 3
Fig. S3	ESI-LCMS data of **M1**	page 4
Fig. S4	^{1}H and ^{13}C-NMR-spectra of compound **2**	page 5
Fig. S5	ESI-LCMS data of compound **2**	page 6
Fig. S6	^{1}H and ^{13}C-NMR-spectra of compound **3**	page 7
Fig. S7	^{1}H and ^{13}C-NMR-spectra of compound **M2**	page 8
Fig. S8	ESI-LCMS data of compound **M2**	page 9
Fig. S9	^{1}H and ^{13}C-NMR-spectra of compound **M3**	page 10
Fig. S10	ESI-LCMS data of compound **M3**	page 11
Fig. S11	Single versus combined addition of **Olaparib** and **Cpd1**	page 12

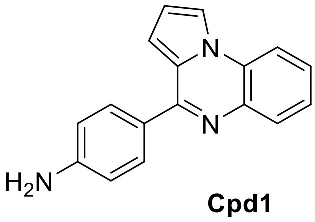

Cpd1

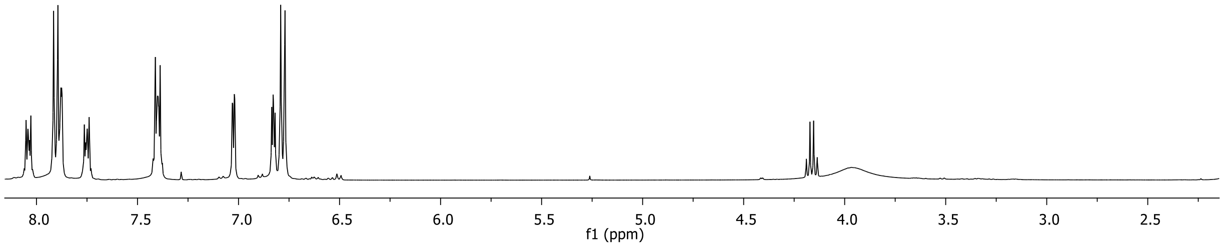

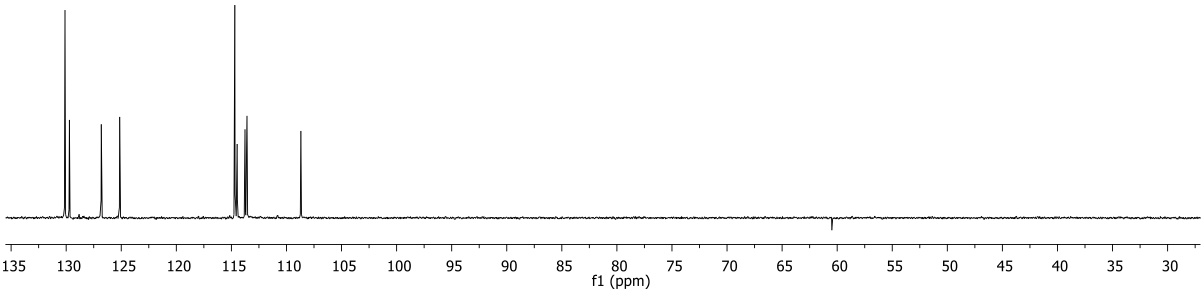

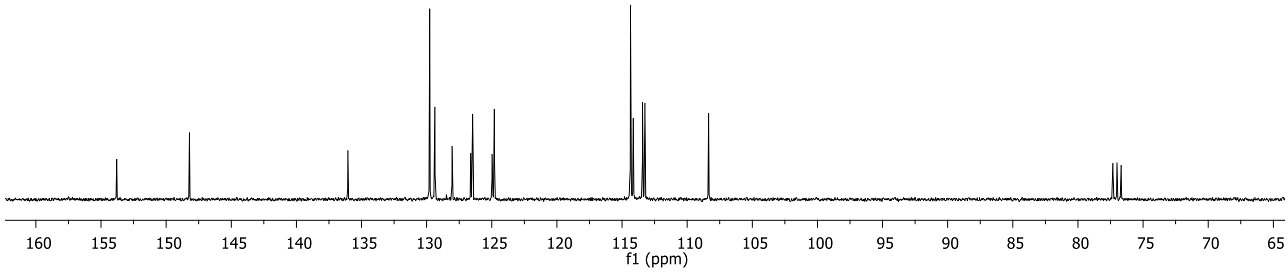

Fig. S1. ^{1}H (400 MHz, $CDCl_3$), DEPT-135, and ^{13}C (101 MHz) NMR spectra of **Cpd1**.

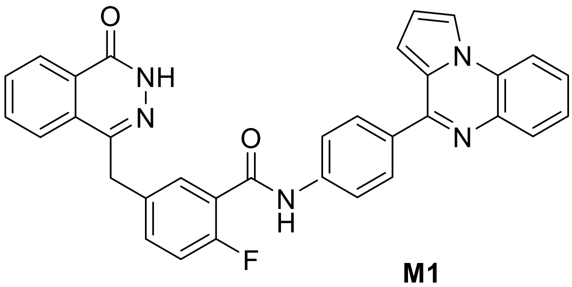

M1

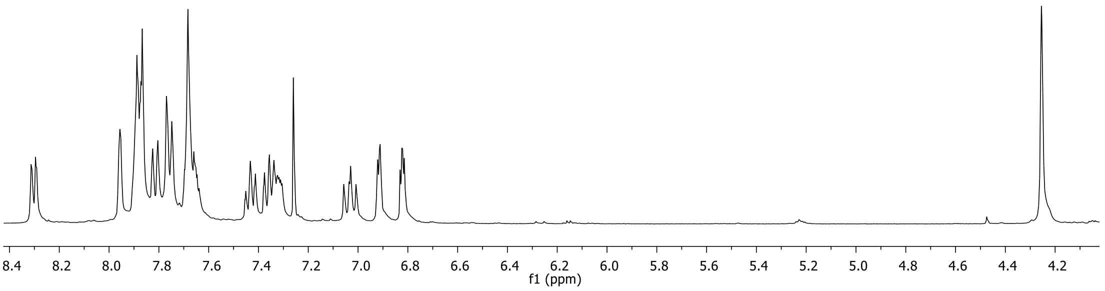

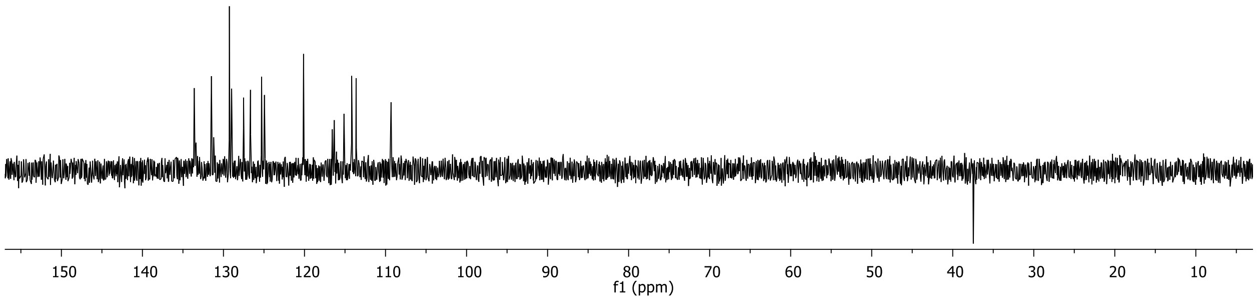

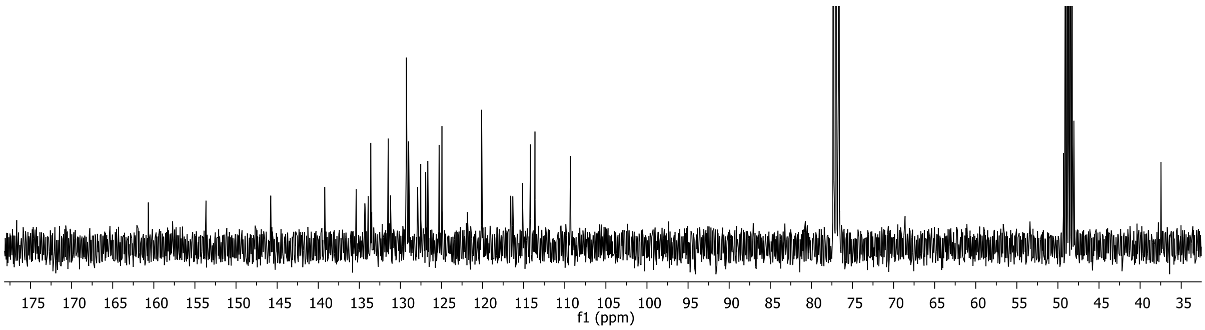

Fig. S2. ^{1}H (400 MHz, CDCl3, DMSO-*d6)*, DEPT-135, and ^{13}C (101 MHz) NMR spectra of **M1**.

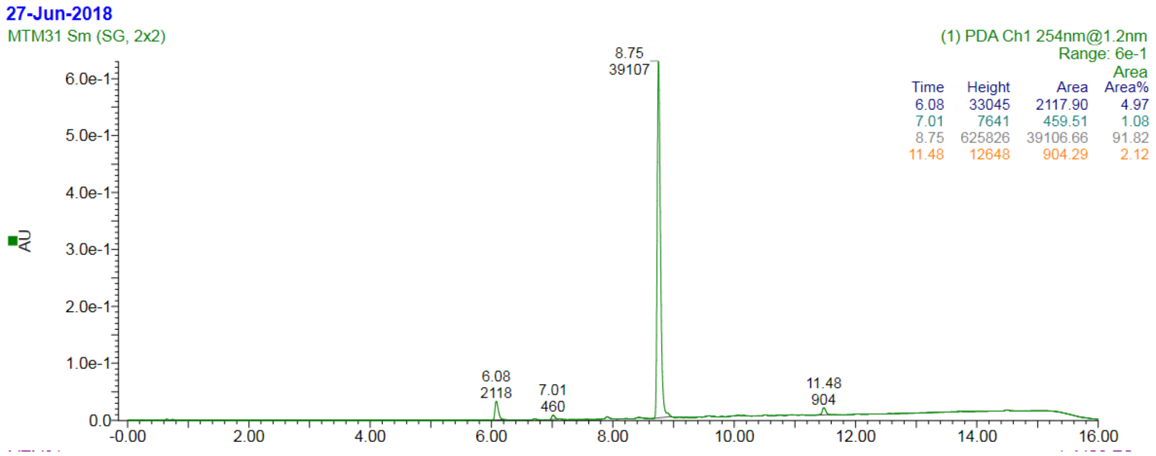

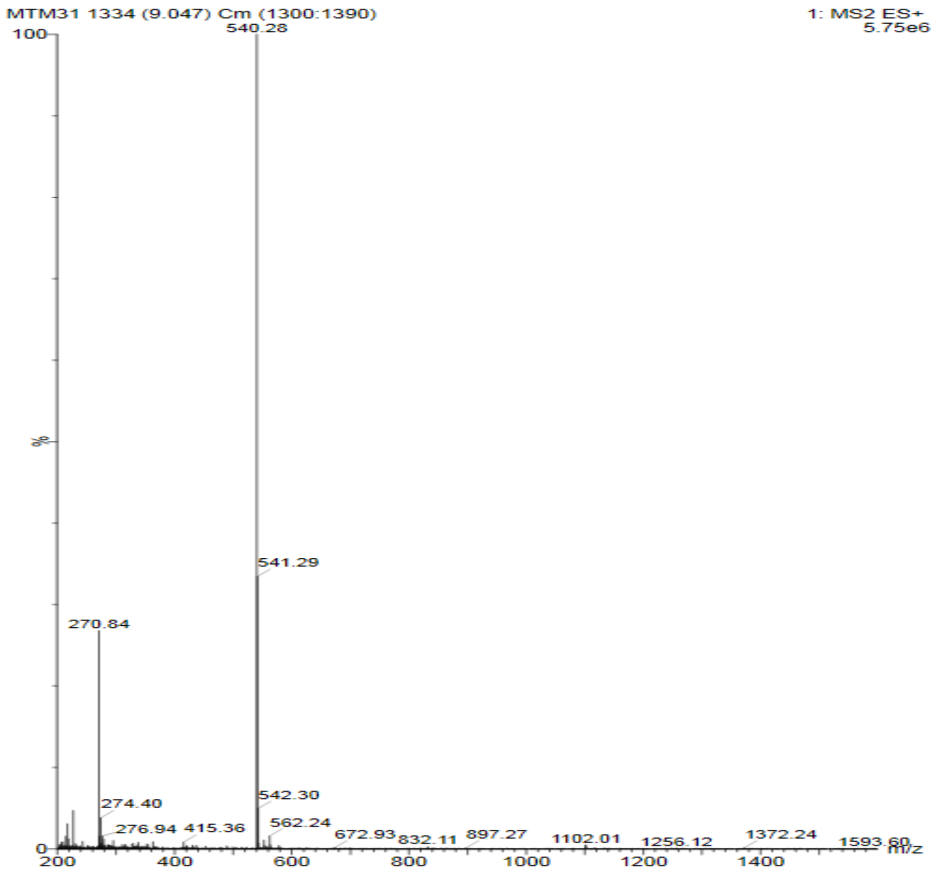

Fig. S3. ESI-LCMS data of compound **M1**.

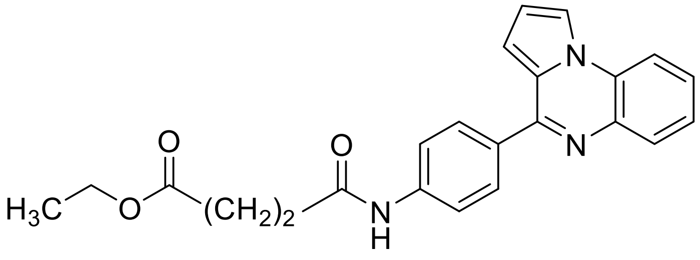

2

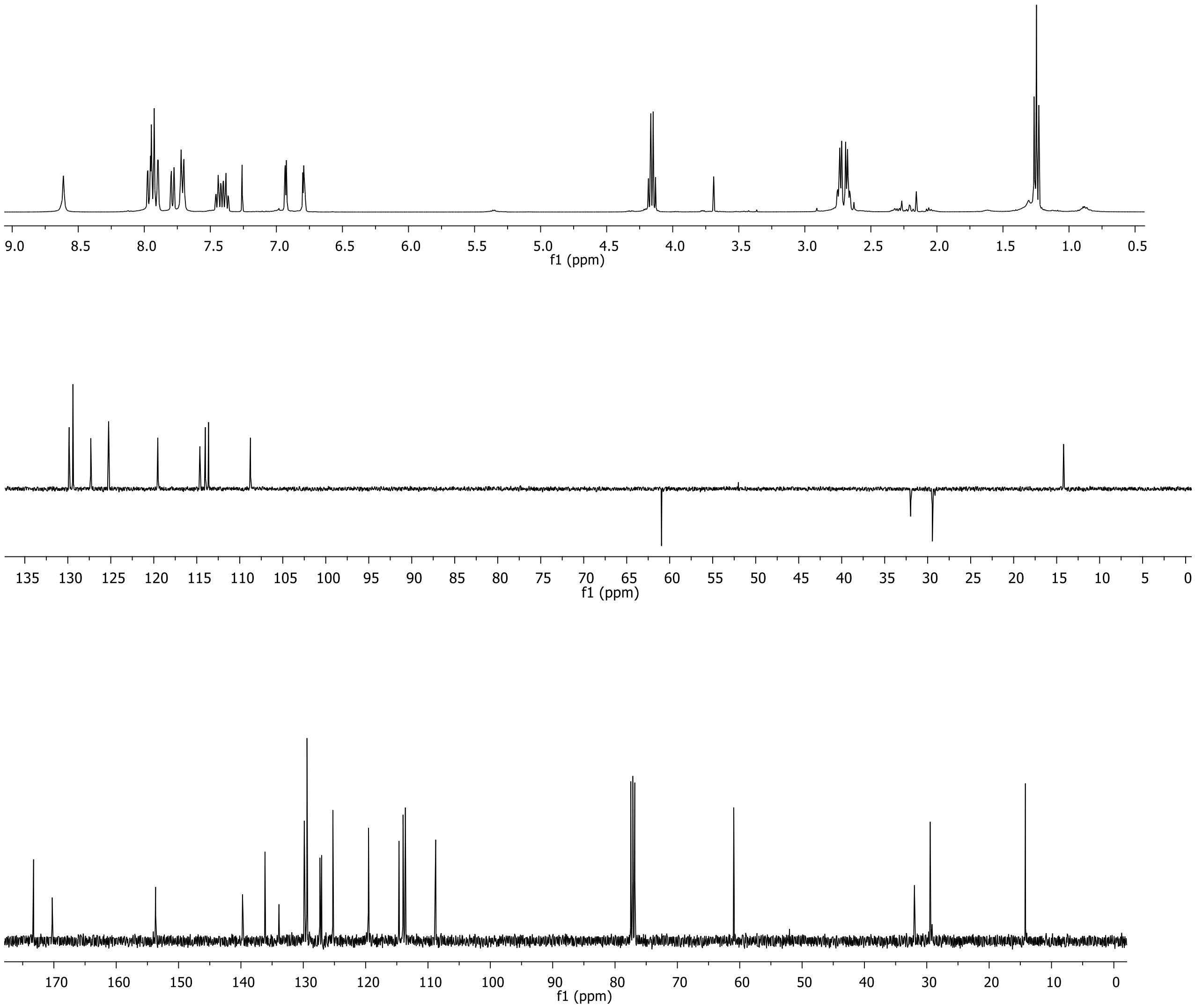

Fig. S4. ^{1}H (400 MHz, $CDCl_3$), DEPT-135, and ^{13}C (101 MHz) NMR-spectra of compound **2.**

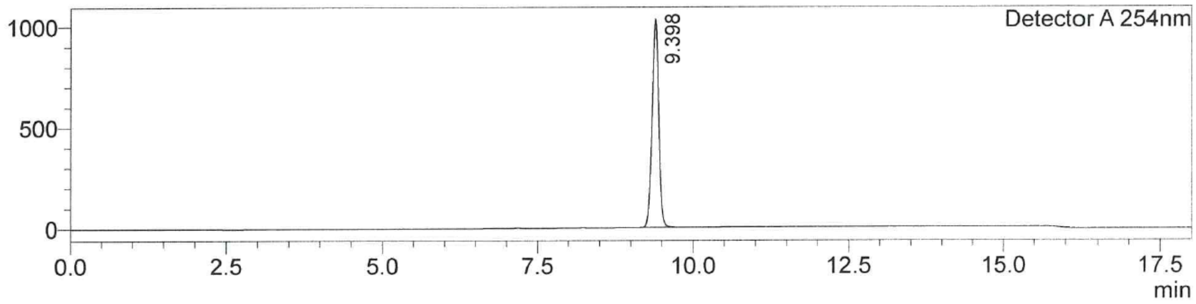

Detector A 254nm

Peak#	Ret. Time	Area	Height	Area%
1	9.398	7577012	1026292	100.000
Total		7577012	1026292	100.000

Line#:1 R.Time:9.760(Scan#:5857)
MassPeaks:370
Spectrum Mode:Averaged 9.757-9.763(5855-5859) Base Peak:416.05(2372151)
BG Mode:Calc Segment 1 - Event 1

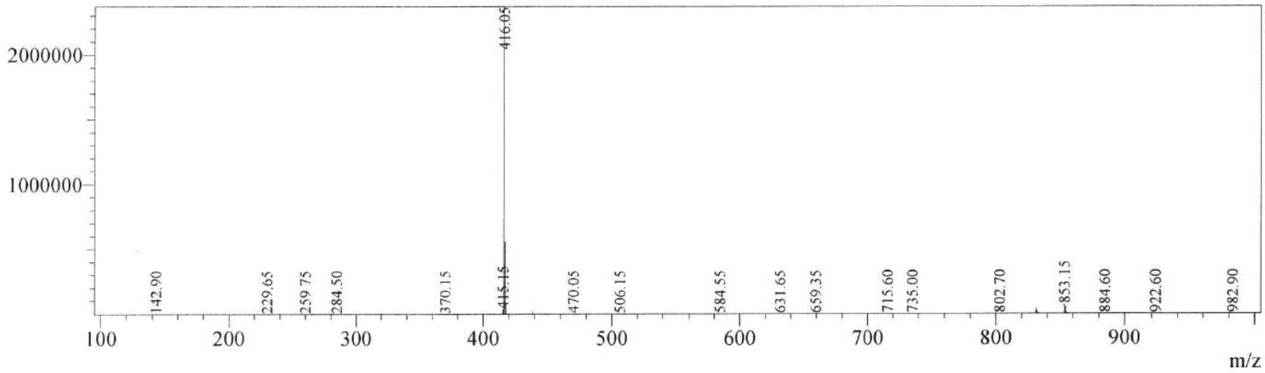

Fig. S5. ESI-LCMS data of compound **2**.

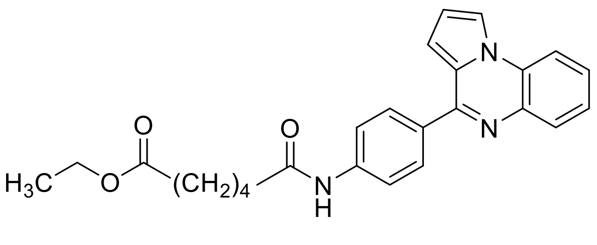

3

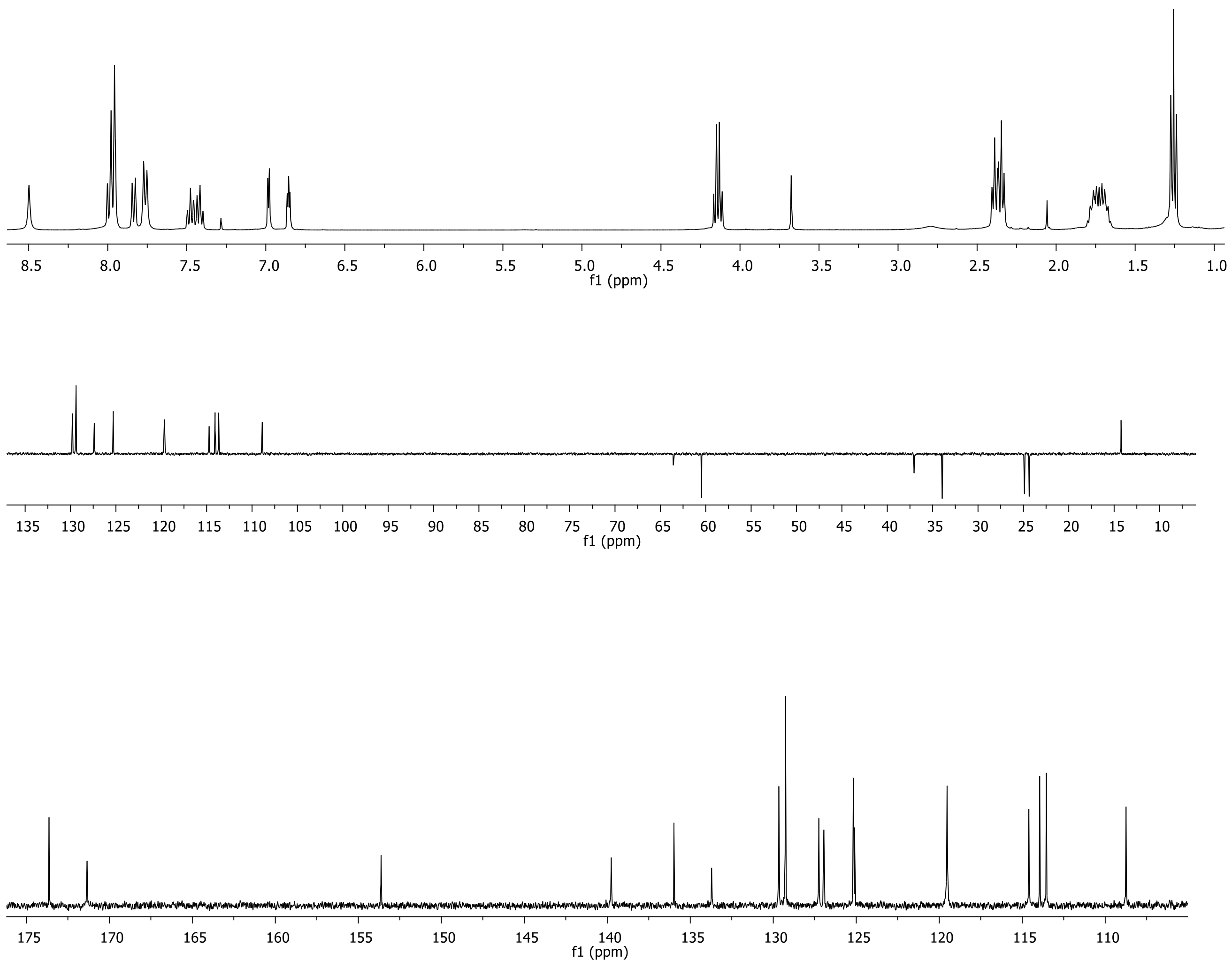

Fig. S6. ¹H (400 MHz, $CDCl_3$), DEPT-135, and ¹³C (101 MHz) NMR-spectra of compound **3.**

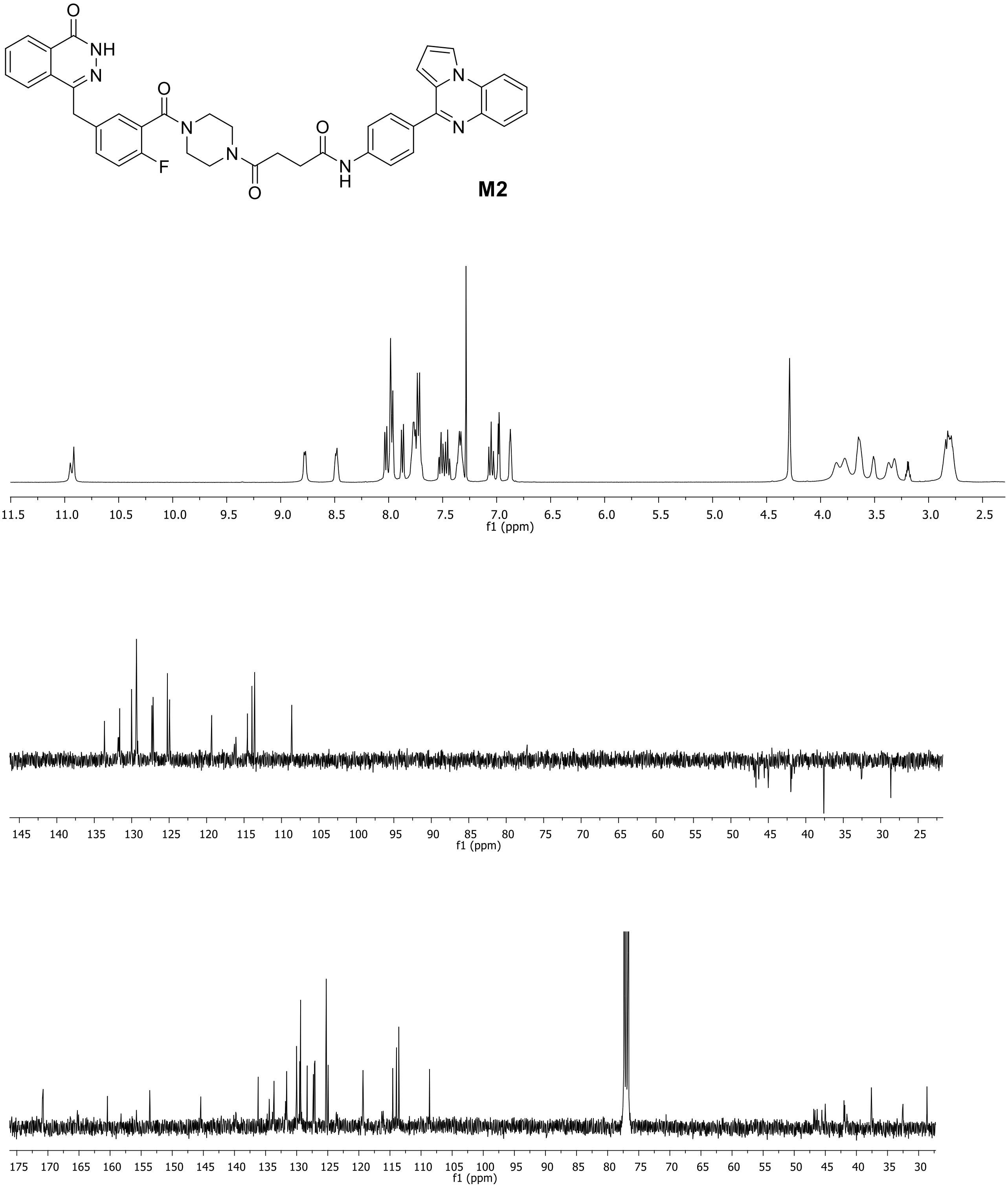

Fig. S7. ^{1}H (400 MHz, $CDCl_3$), DEPT-135, and ^{13}C (101 MHz) NMR-spectra of compound **M2.**

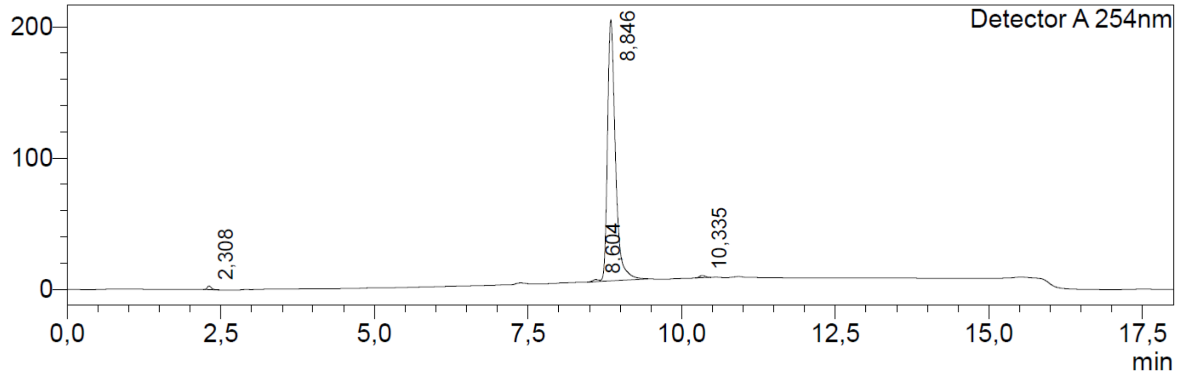

Peak#	Ret. Time	Area	Area%
1	2,308	12416	0,686
2	8,604	10964	0,606
3	8,846	1774504	98,087
4	10,335	11226	0,621
Total		1809111	100,000

Spectrum Mode:Averaged 9,240-9,247(5545-5549) Base Peak:354,65(3126175)
BG Mode:Calc Segment 1 - Event 1

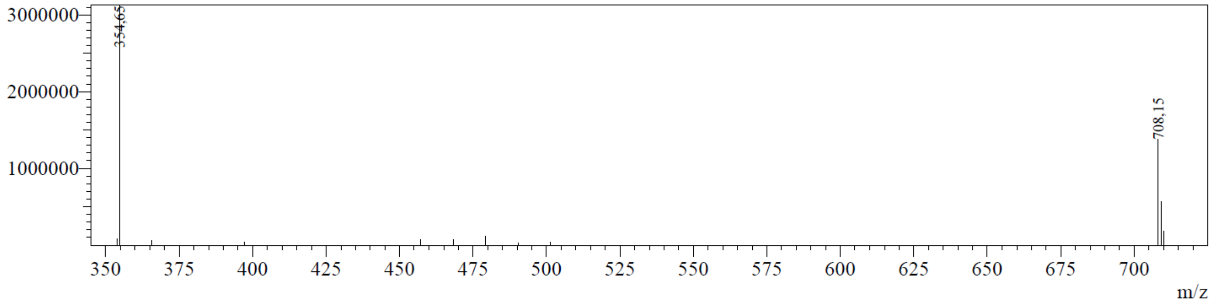

Fig. S8. ESI-LCMS data of compound **M2.**

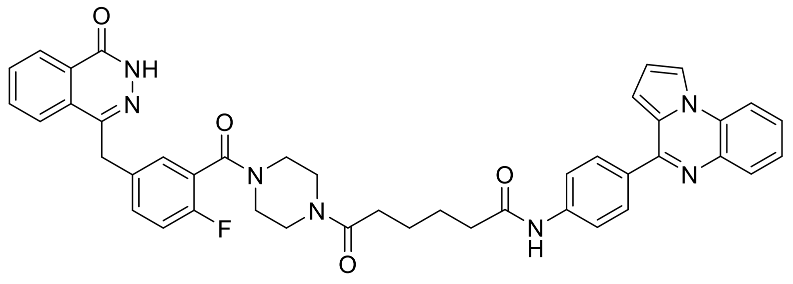

M3

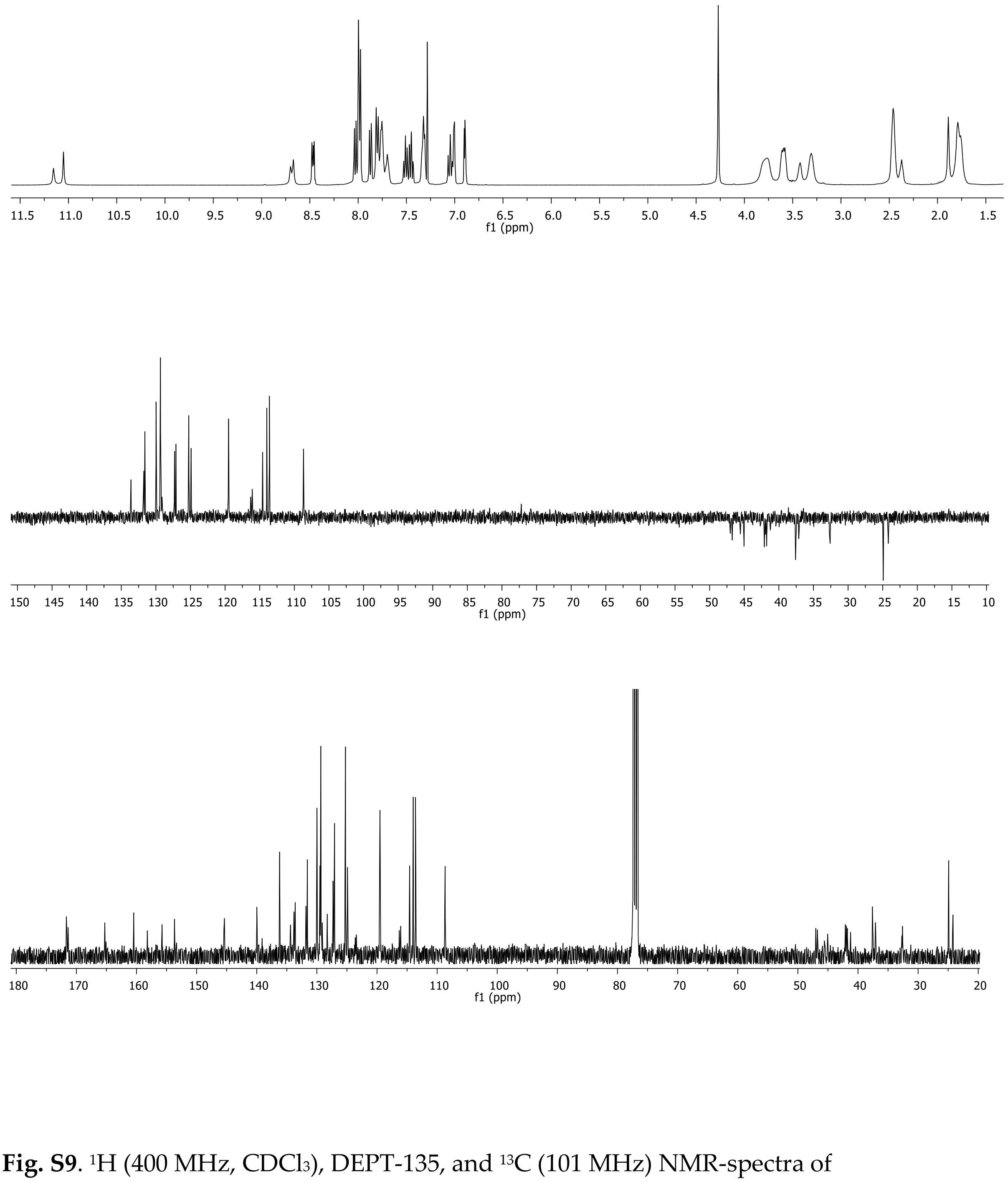

Fig. S9. ^{1}H (400 MHz, $CDCl_3$), DEPT-135, and ^{13}C (101 MHz) NMR-spectra of compound **M3.**

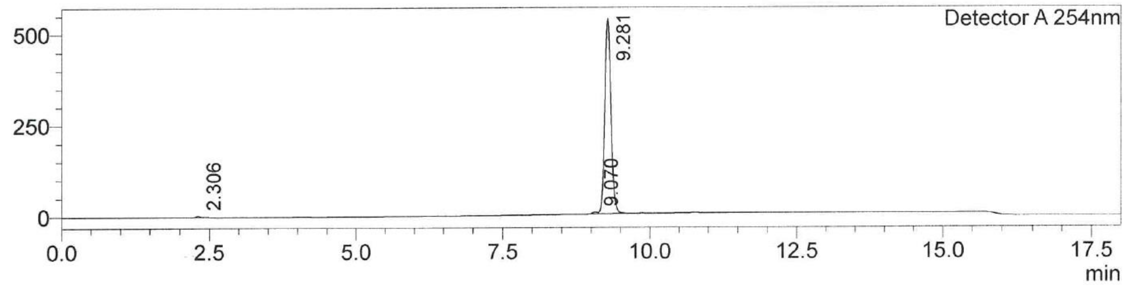

Detector A 254nm

Peak#	Ret. Time	Area	Height	Area%
1	2.306	16976	3557	0.432
2	9.070	32717	5068	0.833
3	9.281	3877391	533742	98.735
Total		3927083	542367	100.000

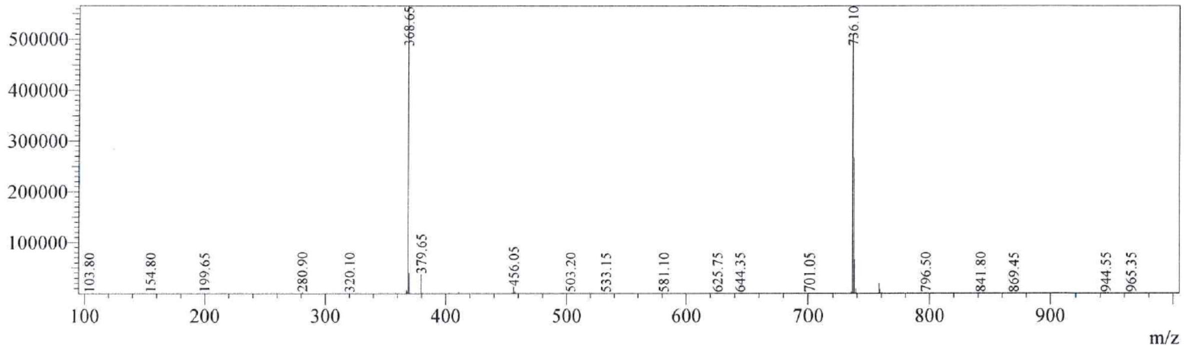

Fig. S10. ESI-LCMS data of compound **M3.**

(a)

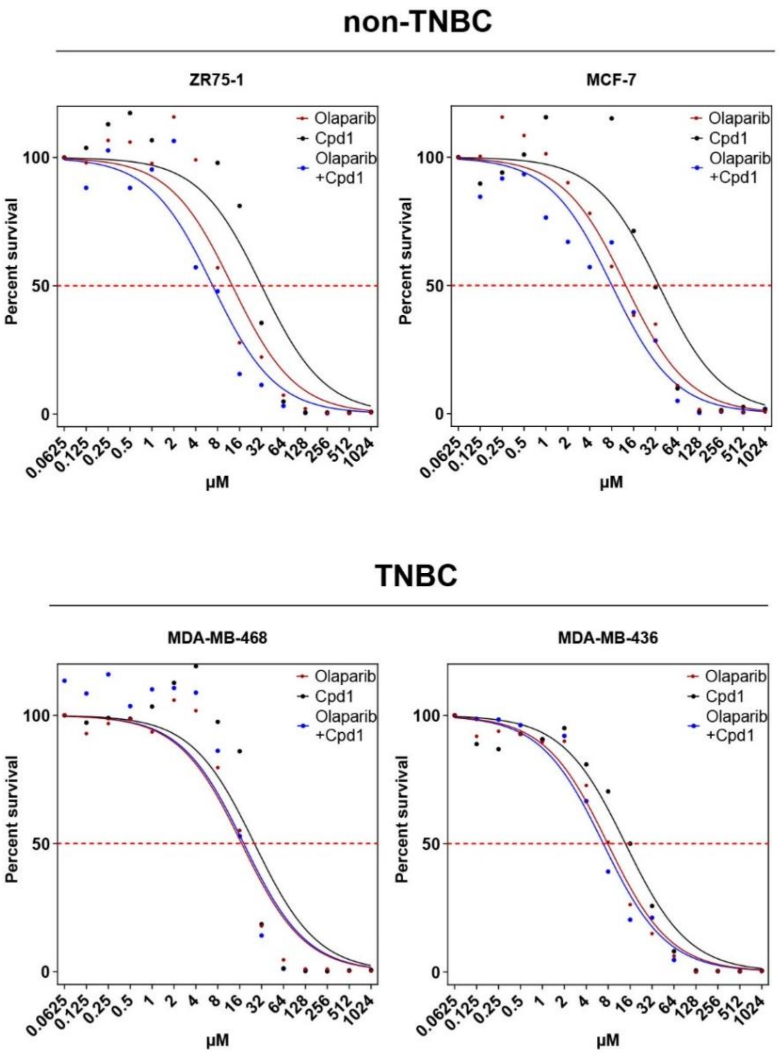

(b)

Cell line	IC_{50} [μM]		
	Olaparib	Cpd1	Olaparib + Cpd1
ZR75-1	12.71	31.98	6.83
MCF-7	12.47	35.60	8.05
MDA-MB-468	17.03	25.51	18.19
MDA-MB-436	7.88	13.79	6.85

Fig. S11: Survival after single versus combined addition of **Olaparib** and **Cpd1**.

Non-TNBC cell lines ZR75-1 and MCF-7 as well as TNBC cell lines MDA-MB-468 and MDA-MB-436 were treated with **Olaparib** (red), **Cpd1** (black) and **Olaparib** plus **Cpd1** (blue). **(a)** Survival curves were generated by GraphPad9 following MTT analyses. Each data point represents the mean of four values from two independent experiments. (**b**) IC_{50} values were calculated from survival curves displayed in (**a**) by use of GraphPad Prism 9. Statistical analyses of the data are shown in **Supplementary Tables**.

Supplementary Material

Dual PARP1 and RAD51 Inhibitory Drug Conjugates Show Synergistic and Selective Effects on Breast Cancer Cells

Matthews M. Malka[1#], Julia Eberle[2#], Kathrin Niedermayer[2], Darius P. Zlotos[1*§] and Lisa Wiesmüller[2*§]

[1]Department of Pharmaceutical Chemistry, The German University in Cairo, New Cairo City, Main Entrance of Al Tagamoa Al Khames, 11835 Cairo, Egypt; darius.zlotos@guc.edu.eg
[2]Department of Obstetrics and Gynecology, Ulm University, Prittwitzstrasse 43, 89075 Ulm, Germany; lisa.wiesmueller@uni-ulm.de

Supplementary Tables

Legend to Supplementary Tables ad Figure 2A and Table 2 page 2
Statistics - comparison of cell lines page 3-4
Statistics - comparison of treatments page 5-6

Legend to Supplementary Tables ad Supplementary Figure 11 page 7
Statistics - comparison of treatments page 8

Supplementary Tables ad Figure 2A and Table 2. Statistical comparisons for single and dual inhibitory compound treatments.

For statistical analysis of differences between the IC_{50} values calculated from the survival curves in **Figure 2A** and summarized in **Table 2** we used GraphPad8 (GraphPad Software, San Diego, California, USA). Statistical significance was determined using Extra sum of-squares F-test, nonlin fit.

Statistics - comparison of cell lines

Olaparib

	ZR75-1	MCF-7	MDA-MB-453	MDA-MB-468	HCC-1937	MDA-MB-436
ZR75-1	x	0.1439	<0.0001	0.0032	0.2293	<0.0001
MCF-7	0.1439	x	<0.0001	0.1899	0.7174	<0.0001
MDA-MB-453	<0.0001	<0.0001	x	0.0018	<0.0001	<0.0001
MDA-MB-468	0.0032	0.1899	0.0018	x	0.0685	<0.0001
HCC-1937	0.2293	0.7174	<0.0001	0.0685	x	<0.0001
MDA-MB-436	<0.0001	<0.0001	<0.0001	<0.0001	<0.0001	x

M1

	ZR75-1	MCF-7	MDA-MB-453	MDA-MB-468	HCC-1937	MDA-MB-436
ZR75-1	x	<0.0001	0.0075	0.0984	0.7089	<0.0001
MCF-7	<0.0001	x	0.7753	0.0158	0.0019	<0.0001
MDA-MB-453	0.0075	0.7753	x	0.168	0.0183	<0.0001
MDA-MB-468	0.0984	0.0158	0.168	x	0.1544	<0.0001
HCC-1937	0.7089	0.0019	0.0183	0.1544	x	<0.0001
MDA-MB-436	<0.0001	<0.0001	<0.0001	<0.0001	<0.0001	x

M2

	ZR75-1	MCF-7	MDA-MB-453	MDA-MB-468	HCC-1937	MDA-MB-436
ZR75-1	x	0.4884	<0.0001	<0.0001	<0.0001	<0.0001
MCF-7	0.4884	x	<0.0001	<0.0001	<0.0001	<0.0001
MDA-MB-453	<0.0001	<0.0001	x	<0.0001	<0.0001	<0.0001
MDA-MB-468	<0.0001	<0.0001	<0.0001	x	0.7752	0.0004
HCC-1937	<0.0001	<0.0001	<0.0001	0.7752	x	0.0171
MDA-MB-436	<0.0001	<0.0001	<0.0001	0.0004	0.0171	x

M3

	ZR75-1	MCF-7	MDA-MB-453	MDA-MB-468	HCC-1937	MDA-MB-436
ZR75-1	x	0.3411	0.2055	0.0001	<0.0001	<0.0001
MCF-7	0.3411	x	0.05	0.0262	<0.0001	<0.0001
MDA-MB-453	0.2055	0.05	x	<0.0001	<0.0001	<0.0001
MDA-MB-468	0.0001	0.0262	<0.0001	x	<0.0001	<0.0001
HCC-1937	<0.0001	<0.0001	<0.0001	<0.0001	x	<0.0001
MDA-MB-436	<0.0001	<0.0001	<0.0001	<0.0001	<0.0001	x

Cpd1

	ZR75-1	MCF-7	MDA-MB-453	MDA-MB-468	HCC-1937	MDA-MB-436
**ZR75-1**	x	0.122	0.3224	0.0053	0.2893	0.0008
**MCF-7**	0.122	x	0.5777	0.1281	0.0035	0.0754
**MDA-MB-453**	0.3224	0.5777	x	0.0499	0.0216	0.0178
**MDA-MB-468**	0.0053	0.1281	0.0499	x	<0.0001	0.9013
**HCC-1937**	0.2893	0.0035	0.0216	<0.0001	x	<0.0001
**MDA-MB-436**	0.0008	0.0754	0.0178	0.9013	<0.0001	x

Statistics - comparison of treatments

ZR75-1

	Olaparib	M1	M2	M3	Cpd1
**Olaparib**	x	<0.0001	<0.0001	0.0002	0.1305
**M1**	<0.0001	x	<0.0001	<0.0001	<0.0001
**M2**	<0.0001	<0.0001	x	0.7778	0.0644
**M3**	0.0002	<0.0001	0.7778	x	0.0585
**Cpd1**	0.1305	<0.0001	0.0644	0.0585	x

MCF-7

	Olaparib	M1	M2	M3	Cpd1
**Olaparib**	x	<0.0001	0.1538	0.3822	0.026
**M1**	<0.0001	x	<0.0001	<0.0001	<0.0001
**M2**	0.1538	<0.0001	x	0.8131	0.6469
**M3**	0.3822	<0.0001	0.8131	x	0.5172
**Cpd1**	0.026	<0.0001	0.6469	0.5172	x

MDA-MB-453

	Olaparib	M1	M2	M3	Cpd1
**Olaparib**	x	<0.0001	<0.0001	0.0503	0.2007
**M1**	<0.0001	x	<0.0001	<0.0001	<0.0001
**M2**	<0.0001	<0.0001	x	0.0085	<0.0001
**M3**	0.0503	<0.0001	0.0085	x	0.0138
**Cpd1**	0.2007	<0.0001	<0.0001	0.0138	x

MDA-MB-468

	Olaparib	M1	M2	M3	Cpd1
**Olaparib**	x	<0.0001	<0.0001	0.0014	0.0049
**M1**	<0.0001	x	<0.0001	<0.0001	<0.0001
**M2**	<0.0001	<0.0001	x	<0.0001	<0.0001
**M3**	0.0014	<0.0001	<0.0001	x	<0.0001
**Cpd1**	0.0049	<0.0001	<0.0001	<0.0001	x

HCC-1937

	Olaparib	M1	M2	M3	Cpd1
**Olaparib**	x	<0.0001	<0.0001	<0.0001	0.5868
**M1**	<0.0001	x	<0.0001	<0.0001	<0.0001
**M2**	<0.0001	<0.0001	x	0.1187	<0.0001
**M3**	<0.0001	<0.0001	0.1187	x	<0.0001
**Cpd1**	0.5868	<0.0001	<0.0001	<0.0001	x

MDA-MB-436

	Olaparib	M1	M2	M3	Cpd1
**Olaparib**	x	<0.0001	<0.0001	<0.0001	<0.0001
**M1**	<0.0001	x	<0.0001	<0.0001	0.4928
**M2**	<0.0001	<0.0001	x	0.1297	<0.0001
**M3**	<0.0001	<0.0001	0.1297	x	<0.0001
**Cpd1**	<0.0001	0.4928	<0.0001	<0.0001	x

Supplementary Tables ad Supplementary Figure 11. Statistical comparison for single and combined drug treatments.

For statistical analysis of differences between the IC_{50} values calculated from the survival curves in **Supplementary Figure 11** we used GraphPad9 (GraphPad Software, San Diego, California, USA). Statistical significance was determined using Extra sum of-squares F-test, nonlin fit.

Statistics - comparison of treatments

ZR75-1

	Olaparib	Cpd1	Olaparib + Cpd1
**Olaparib**	x	0.0001	0.0019
**Cpd1**	0.0001	x	<0.0001
**Olaparib + Cpd1**	0.0019	<0.0001	x

MCF-7

	Olaparib	Cpd1	Olaparib + Cpd1
**Olaparib**	x	0.0001	0.0386
**Cpd1**	0.0001	x	<0.0001
**Olaparib + Cpd1**	0.0386	<0.0001	x

MDA-MB-468

	Olaparib	Cpd1	Olaparib + Cpd1
**Olaparib**	x	0.0306	0.7112
**Cpd1**	0.0306	x	0.1035
**Olaparib + Cpd1**	0.7112	0.1035	x

MDA-MB-436

	Olaparib	Cpd1	Olaparib + Cpd1
**Olaparib**	x	<0.0001	0.2205
**Cpd1**	<0.0001	x	<0.0001
**Olaparib + Cpd1**	0.2205	<0.0001	x
